# The cost of persistent alarm: temporal transcriptional reprogramming of macrophages from acute activation to chronic immune suppression by day 11

**DOI:** 10.3389/fimmu.2026.1729108

**Published:** 2026-08-11

**Authors:** Elizabeth A. Mazzio, Andrew S. Barnes, Ramesh B. Badisa, Selina F. Darling-Reed, Karam F. A. Soliman

**Affiliations:** 1 Florida Agricultural and Mechanical University, College of Pharmacy and Pharmaceutical Sciences, Tallahassee, FL, United States; 2 Neuro-Infectious Disease Program, Mt. Sinai Icahn School of Medicine, New York, NY, United States

**Keywords:** cancer, IEX, immune therapies, macrophage, microbes, tolerance

## Abstract

**Introduction:**

Chronic inflammation has long been associated with cancer initiation, yet the mechanisms linking sustained immune activation to an immune-permissive tumor microenvironment remain incompletely defined. Prevailing explanations such as immune exhaustion (IEX) or free radical mediated tissue damage, fail to account for the active state of immune tolerance, a process driven by potent negative feedback loops that systematically suppress host effector responses.

**Methods:**

To address this gap, we developed an *in vitro* model of macrophage tolerance driven by sustained Toll-like receptor 4 (TLR4) activation using microbial-associated molecular patterns (MAMPs). This system captures the full kinetic progression of the immune response, tracking macrophages from a resting baseline, through acute activation at 24 hours, to a chronic tolerant endpoint at 7–11 days. Methodologically, cells were maintained under a continuous media exchange (+/− *E. coli* O111:B4 LPS) featuring high glucose and an elevated volume-to-cell ratio. This setup effectively eliminates autocrine interference and toxic byproducts, successfully isolating the direct consequences of sustained TLR4 signaling across extended durations.

**Results:**

Whole-transcriptome sequencing, validated by RT-PCR and select protein immunoblots, revealed that both “exhaustion” and “tolerance” are mischaracterized. Rather than a passive exhaustion state or a simple trajectory of diminishing returns, the resting-acute-chronic continuum drives a potent, active negative-feedback mechanism across an eight-phase bidirectional trajectory. By days 7–11, macrophages shifted to a TAM-like signature, overexpressing immune checkpoints (PD-L1/MSN, TIM-3, SPP1, CD73, CD44, LILRs) and regulatory suppressive networks (SOCS/JAK/STAT, IL-10, CCL2/7/12, CXCL2), while downregulating classical (H2-D1/K1) and non-classical (H2-Q/T) MHC-I antigen-presenting genes. These alterations coincided with the profound loss of interferon-stimulated genes (ISGs) including the IFIT family, Ly6e, Irf7, Rsad2/Viperin, and the Oas gene family, fundamentally crippling the machinery required for antiviral and antitumor immune surveillance. Moreover, this chronic stage drove the upregulation of degradative proteases (cathepsins, Adam8, S100a8, Klk9, carboxypeptidase D), integrins/adhesion molecules (Itga5, Marcks, Msr1/CD204, Alcam), iron-storage transcripts, lipid translocases (Cd36), and fatty acid-binding proteins. Concurrently, macrophages upregulated Nos2/Cox2 alongside the metabolic collapse of mitochondrial OXPHOS genes and Acod1 (itaconate). Uniquely, this negative feedback loop coincided with a sustained, massive surge in a cluster of poorly characterized small proline-rich proteins (SPRRs), specifically Sprr2b, 2e, 2d, 2f, 2g, 2h, 2i, 2j, and 2k.

**Discussion:**

Overall, these results indicate that chronic inflammatory signaling can ultimately trigger a profound coordinated negative-feedback program consistent with a reduced immune recognition and defense pathway signatures. Ultimately, this study provides a reproducible *in vitro* macrophage model to investigate immune suppression. It offers deeper insights into how chronic inflammation impairs host defenses against viral and tumor cells.

## Introduction

Deciphering how acute inflammation degenerates into chronic immunosuppressive states remains a foundational, yet elusive, challenge with profound implications for oncology and virology. In a balanced immune system, negative feedback pathways serve a vital dual purpose. They suppress active immune responses once a pathogen is cleared to limit tissue injury, while simultaneously safeguarding self-tolerance to protect host tissues from aberrant attack. Mechanistically, the acute response begins when host pattern recognition receptors (PRRs) engage microbial signatures MAMPs (Microbe-Associated Molecular Patterns) which alert the body’s immune system to the presence of microbes. While this acute cascade effectively primes adaptive immunity; a principle leveraged by MAMP-based vaccine adjuvants, its application in cancer immunotherapy has achieved limited success, as repeated exposure inevitably steers the system toward a state of induced tolerance.

This modern reliance on microbial components to trigger an antitumor host response traces its origins to the late nineteenth and early twentieth centuries. During this era, pioneering clinicians such as Wilhelm Busch, Friedrich Fehleisen, and William Coley first demonstrated that injections of bacterial mixtures—rich in what are now recognized as innate MAMPs—could provoke systemic immune responses capable of shrinking inoperable tumors (1). However, despite the historical development of “Coley’s Toxins” (composed of heat-killed Streptococcus pyogenes and Serratia marcescens) and the contemporary deployment of advanced MAMP-based regimens—including Bacillus Calmette–Guérin (BCG), lipopolysaccharide (LPS) analogues, and Immunomax—these therapies continue to yield only modest clinical success (2, 3) (4–7).

These clinical limitations stem primarily from the onset of tolerance following sustained antigenic stimulation. Rather than maintaining an active defense, repeated MAMP exposure drives a compensatory negative feedback cascade that phenocopies the immune suppression seen in chronic inflammatory states.

Because this transition remains a predominantly descriptive phenomenon, there is an urgent need to map the exact cellular mechanisms driving this desensitization. Resolving this enigma is critical, as it directly dictates the therapeutic efficacy of a wide array of conventional tumor immunotherapies (8–14), as well as MAMP-based regimens (14–16).

Crucially, the relevance of this immunosuppressive state extends beyond therapeutic failure; it represents a foundational mechanism in cancer initiation, where the transition from acute to chronic inflammation establishes a microenvironment highly conducive to tumor development. Mechanistically, persistent tissue exposure to MAMPs or age-related damage-associated molecular patterns (DAMPs) eventually shifts the microenvironment by inducing and recruiting an array of suppressive immune cells. This tolerogenic influx includes regulatory T cells (Tregs), myeloid-derived suppressor cells (MDSCs), tumor-associated neutrophils (TANs), and tumor-associated macrophages (TAMs), and CD4^+^CD25^+^Foxp3^+^ cells, which collectively establish a protective niche where tumors can thrive (17–24).

Given that macrophages are central drivers and coordinators of this networked immune tolerance—and are among the first cells to sense MAMPs and DAMPs via Toll-like receptors (TLRs) and NOD-like receptors (NLRs)—we developed a time-lapse *in vitro* model of prolonged macrophage TLR4 activation. This highly controlled system allows us to dissect the precise kinetics and molecular shifts that define immune tolerance (9, 25).

## Methods

### Cell culture

Mouse macrophage RAW 264.7 cells (RRID: CVCL_0493) were obtained from the American Type Culture Collection (ATCC, Manassas, VA, USA). Cells were maintained in high-glucose DMEM (4,500 mg/L) supplemented with 5% FBS, 4 mM L-glutamine, and penicillin/streptomycin (100 U/0.1 mg/mL) at 37°C in a humidified 5% CO_2_ atmosphere. For experiments, cells were subcultured into DMEM without phenol red (4,500 mg/L glucose) containing 5% FBS, 4 mM L-glutamine, and penicillin/streptomycin (100 U/0.1 mg/mL). Lipopolysaccharide (LPS) from *Escherichia coli* O111:B4 was used in all experiments at a final working concentration of 500 ng/mL.

### Endotoxin tolerance induction method validation.

A pre-validation study was performed to establish culture conditions that prevented the accumulation of toxic cellular byproducts during prolonged LPS exposure. First a dose was chosen from RAW 264.7 macrophages seeded at 100,000 cells in high glucose media in 96-well plates (200 µL/well) where 500 ng/mL LPS was sufficient to produce a strong response and no toxicity (**Supplementary File 3**). A second study was performed in RAW264.7 macrophages with initial cell densities (234–439,000 cells per well) in 200 µL high-glucose DMEM in 96-well plates and stimulated with 500 ng/mL LPS for 18 hours. Optimal plating density with meager NO accumulation in the media, no cell death and minimal use of glucose for long term tolerance studies was set at approximately 40,000 cells per well in 200 µL medium (<200,000 cells/mL). Cell death appeared to correlate more closely with glucose depletion at high cell densities rather than nitric oxide production or immune activation alone. (**Supplementary File 3**). This condition was selected for extrapolation to flask-scale experiments (75 cm^2^).

**Figure 1:**
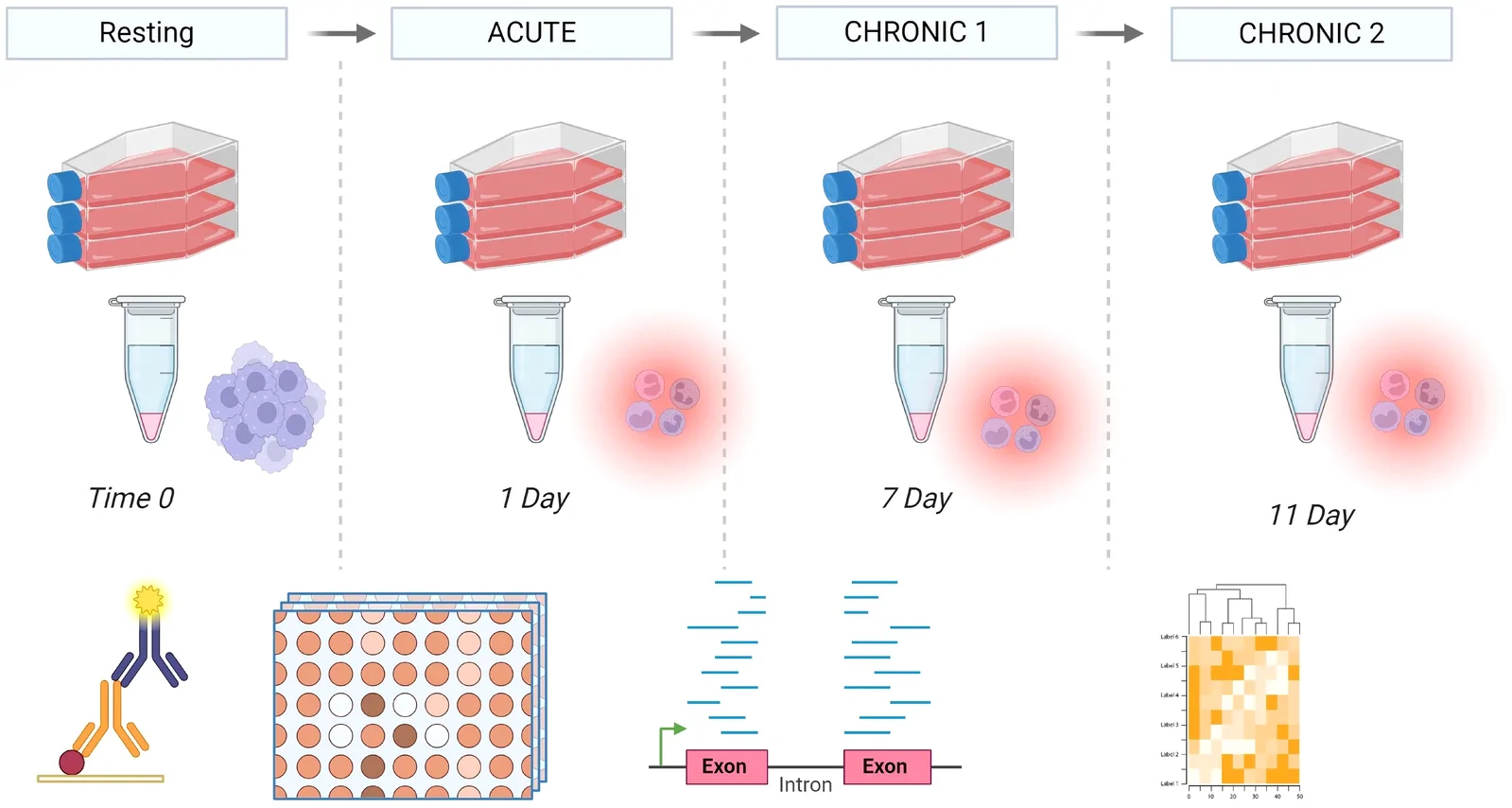
Experimental workflow for sustained LPS stimulation. RAW 264.7 macrophages were collected at resting (0 h), acute (24 h), and chronic (7 and 11 days) time points for phenotype transition analysis by transcriptomic and proteomic profiling.

Based on this method validation, four experimental conditions were established for the main study (**Figure 1**). Baseline untreated control cells were cultured in the absence of LPS, while acute treatment groups were exposed to 500 ng/mL LPS for 24 hours. For chronic treatment groups, cells were continuously maintained with complete media/LPS replacement every 18 hours to prevent glucose depletion, accumulation of toxic metabolic byproducts, and self-amplifying cytokine feedback while maintaining sustained receptor-level stimulation. Supernatants and cells were collected at the 0- and 24-hour time points. For the 7- and 11-day treatment groups, cells and supernatants were collected following the final 18-hour treatment cycle and stored at −80°C for downstream transcriptomic and protein analyses. Cell viability was maintained throughout the study, and low-density culture conditions prevented detectable nitric oxide accumulation over the course of the experiment.

### Nitric oxide monitoring

Nitrite (NO_2_^–^) levels were quantified using the Griess assay. The Griess reagent was prepared by mixing equal volumes of 1.0% sulfanilamide in 10% phosphoric acid and 0.1% N-(1-naphthyl)ethylenediamine in deionized water. This reagent was added directly to sample supernatants (DMEM without phenol red), and samples were incubated at room temperature for 10 min. Absorbance was measured at 540 nm using a Synergy HTX multimode reader (BioTek, Winooski, VT, USA).

### RNA sequencing

RNA sequencing was performed in collaboration with LC Biosciences (Houston, TX, USA). Total RNA was extracted using TRIzol reagent (Thermo Fisher, cat. 15596018) following the manufacturer’s instructions. RNA quality and quantity were assessed using the Bioanalyzer 2100 and the RNA 6000 Nano LabChip Kit (Agilent, cat. 5067-1511). Only samples with an RNA integrity number (RIN) greater than 7.0 were used for library construction. Poly(A) mRNA was purified from 5 µg of total RNA using Dyn beads Oligo(dT) (Thermo Fisher) with two rounds of selection. Purified mRNA was fragmented at 94°C for 5–7 min using the Magnesium RNA Fragmentation Module (NEB, cat. E6150). The resulting RNA fragments were reverse transcribed into cDNA using SuperScript II Reverse Transcriptase (Invitrogen, cat. no. 1896649). U-labeled second-strand cDNA synthesis was carried out with *E. coli* DNA polymerase I (NEB, cat. M0209), RNase H (NEB, cat. M0297), and dUTP solution (Thermo Fisher, cat. R0133). A-tailing was performed to prepare blunt-ended fragments for adapter ligation. Indexed dual adapters with a T-overhang were ligated, and size selection was performed with AMPure XP beads. U-labeled second-strand cDNAs were treated with heat-labile UDG (NEB, cat. M0280) before amplification. Libraries were PCR-amplified under the following conditions: initial denaturation at 95°C for 3 min; 8 cycles of 98°C for 15 s, 60°C for 15 s, and 72°C for 30 s; and final extension at 72°C for 5 min. The average insert size of the final cDNA libraries was 300 ± 50 bp. Paired-end sequencing (2 × 150 bp, PE150) was performed on an Illumina NovaSeq 6000 platform according to the manufacturer’s protocol.

### Bioinformatics analysis

Read processing and quality control: A cDNA library was constructed from pooled murine RNA and sequenced on the Illumina NovaSeq 6000 platform using a paired-end RNA-seq approach (2 × 150 bp). Sequencing generated approximately one million paired-end reads. Raw reads containing adapters or low-quality bases were filtered using Cutadapt (RRID: SCR_011841, v1.9). The following reads were removed: (1) those containing adapter sequences, (2) polyA or polyG stretches, (3) >5% unknown nucleotides (N), and (4) >20% low-quality bases (Q ≤ 20). Sequence quality was assessed using FastQC (RRID: SCR_014583, version 0.11.9), which included metrics for Q20, Q30, and GC content. After filtering, high-quality clean reads were retained for downstream analysis (26, 27).

#### Genome alignment

Clean reads were aligned to the mouse reference genome using HISAT2 (RRID: SCR_015530, v2.0.4). HISAT2 discards low-quality reads and maps the remainder to the reference genome, allowing up to 20 multiple alignments per read and a maximum of two mismatches. The software builds a database of potential splice junctions and validates them by aligning previously unmapped reads against the putative junction database (28, 29).

#### Quantification of gene abundance and alternative splicing

Mapped reads from each sample were assembled with StringTie (RRID: SCR_016323, v1.3.4d) using default parameters. Transcriptomes from all samples were merged to reconstruct a comprehensive transcriptome with GffCompare (v0.9.8). Expression levels of transcripts were quantified with StringTie and Ballgown, and mRNA abundance was expressed as fragments per kilobase of transcript per million mapped reads (FPKM). Differential gene expression analysis was performed using DESeq2 for group comparisons and edgeR for pairwise comparisons. Genes with a false discovery rate (FDR) <0.05 and an absolute fold change ≥2 were considered differentially expressed (29–31). Alternative splicing (AS) events were identified with rMATS (version 4.1.1). Significant AS events were defined by FDR <0.05 and classified as skipped exon (SE), mutually exclusive exon (MXE), alternative 5′ splice site (A5SS), alternative 3′ splice site (A3SS), or retained intron (RI) (32, 33). All samples were run in triplicate. Sequencing data are publicly available in the NIH Gene Expression Omnibus (RRID: SCR_005012) under accession number GSE263901.

### Additional RNA-seq data processing and differential expression analysis (DESeq2)

We began with a vendor-provided transcript-level raw count matrix (mouse, Ensembl transcript IDs) and summarized it to the gene level by mapping transcripts to Ensembl gene IDs and summing counts per gene, retaining annotations (trans_type, gene_id, gene_name). Samples were labeled Control, LPS, T2, T3; one failing sample (T3_3) was excluded *a priori*, leaving T3 n=2. Gene-level differential expression was performed in R/DESeq2 with the design ~ condition, with size factors set by the median-of-ratios method, dispersion estimation and shrinkage, and negative binomial GLMs fit with Wald tests (independent filtering left on). We extracted contrasts LPS vs. Control, T2 vs. Control, T3 vs. Control, LPS vs. T2, LPS vs. T3, and LPS vs. pooled (T2,T3); for the pooled comparison, T2 and T3 were collapsed into a single group (“T23”) and the model was refit as ~ condition collapsed. P values were adjusted across genes by Benjamini–Hochberg FDR; for each contrast, we reported log2 fold change (log2FC; positive = higher in the first group), fold change (FC = 2^log2FC), p value, and FDR, along with baseMean and standard errors. Per-contrast result tables were exported with columns trans_type, gene_id, gene_name, baseMean, log2FoldChange, lfcSE, stat, pvalue, padj, FC, direction, and then merged into a single wide (“horizontal”) matrix with one row per gene and contrast-specific columns; targeted extracts were also produced for a specified gene list. Duplicate symbols mapping to distinct Ensembl IDs were retained as separate rows, and all modeling used raw counts (not TPM/FPKM).

### Quantitative real-time PCR

Total RNA was isolated from cells and reverse-transcribed into complementary DNA (cDNA) according to the manufacturer’s protocol. Gene expression was measured using quantitative real-time PCR (RT-qPCR) with gene-specific primers and SYBR Green detection chemistry. Initial amplification reactions were performed using a Bio-Rad T100 thermal cycler (Bio-Rad Laboratories, Hercules, CA, USA). Quantitative PCR detection was subsequently performed using a Fast-16 real-time qPCR system (Suzhou PreciGenome Ltd., Suzhou, China). Fluorescence was measured during each amplification cycle, and melt curve analysis was performed at the end of the reaction to confirm amplification specificity. Gene expression levels were normalized to the housekeeping gene β-actin (Actb). Relative expression levels were calculated using the 2^-ΔΔCt method, with control samples used as the baseline reference. Results are presented as log_2_ fold change relative to control. All reactions were performed in triplicate. The following primer sequences were used for RT-qPCR amplification:

β-actin (Actb) Forward: AGAGGGAAATCGTGCGTGAC β-actin (Actb) Reverse: CAATAGTGATGACCTGGCCGT Il1a Forward: CGAAGACTACAGTTCTGCCATT Il1a Reverse: GACGTTTCAGAGGTTCTCAGAG/Nos2 Forward: GTTCTCAGCCCAACAAATCAAGA Nos2 Reverse: GTGGACGGGTCGATGTCAC/Ccl7 Forward: TGTCCCTGTCATGCTTCTGG Ccl7 Reverse: GCTGCTGGTGATCCTCTTGT/Cd36 Forward: ATGGGCTGTGATCGGAACTG Cd36 Reverse: GTCTTCCACATAAAGCATGTCTCC.

### Protein semiquantitative analysis: cytokine antibody array and ELISA

Mouse cytokine antibody arrays (RayBiotech, GA, USA; product code AAM-CYT-1000) were used to profile cytokine concentrations in cell culture supernatants. Each experiment was performed in duplicate according to the manufacturer’s instructions. Briefly, antibody-coated membranes were incubated for 30 minutes with 1 mL of blocking buffer, followed by overnight incubation at 4°C with 1 mL of sample supernatant under gentle agitation. Membranes were then washed three times, incubated with 1 mL of biotin-conjugated antibodies for 6 h, washed again, and finally incubated with HRP-conjugated streptavidin for 2 h. Signal detection was performed using a chemiluminescent substrate and analyzed by densitometry with a VersaDoc Imager and Quantity One software (Bio-Rad, Hercules, CA, USA). To validate array results, CCL5 was selected as an orthogonal reference protein, as it was strongly affected by tolerance. Compared with acute stimulation, chronic LPS treatment resulted in a marked reduction, with chronic and resting conditions showing comparable levels. CCL5 was validated using the RayBio^®^ Mouse RANTES (CCL5) ELISA kit, following the manufacturer’s protocol. Cell supernatants from all groups were analyzed at 450 nm with a BioTek Synergy HTX Multimode Reader (Agilent Technologies, Santa Clara, CA, USA).

### Data analysis

The bioinformatics tools used for the analysis of the data in this work required the use of various platforms, including the Kanehisa Laboratories (KEGG) (RRID: SCR_012773) (computational pathways (34, 35), Reactome gene sets (36), DAVID (RRID: SCR_001881) functional ontology (37, 38) and Enricher-KG (39, 40). Basic statistical analysis was performed via GraphPad Prism (RRID: SCR_002798) (version 3.0; GraphPad Software Inc., San Diego, CA, USA), with the significance of differences between the groups assessed via one-way ANOVA, followed by Tukey’s *post hoc* means comparison test or Student’s t-test.

## Results

**Figure 2:**
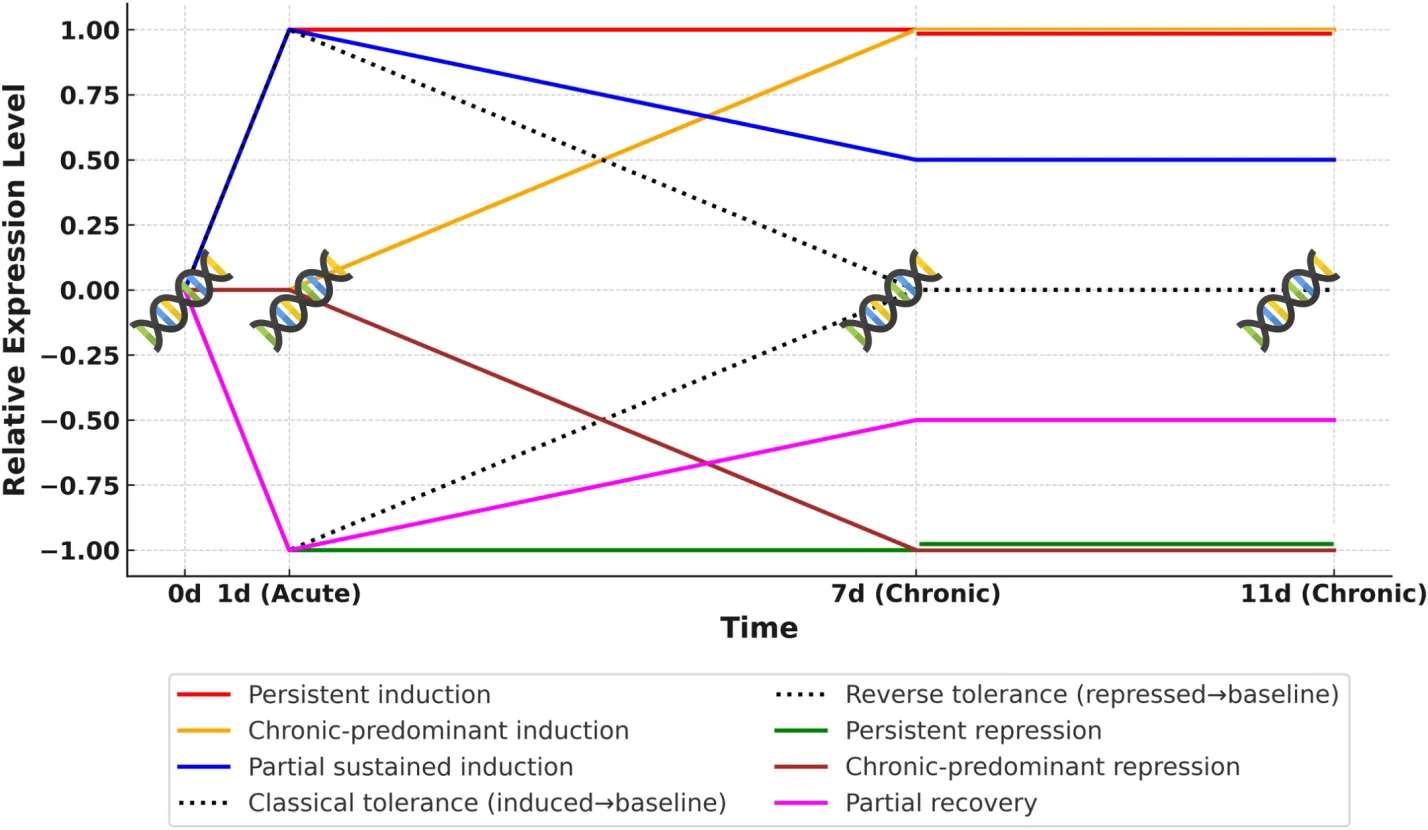
Time-lapse trajectories of gene expression in macrophages under sustained TLR4 stimulation. Responses were resolved into distinct programs spanning across the resting baseline (0 d), acute (1 d), and chronic stimulation (7 and 11 d, which were very similar, suggesting stability of negative feedback). Upregulated trajectories included persistent DEG induction (acute and chronic), chronic-only predominant induction (activation only under prolonged stimulation on days 7, 11), partial tolerance with sustained induction (a strong acute response that declined but remained above the resting baseline), reverse tolerance (acute suppression rebounding to baseline in chronic). Downregulated trajectories included partial reverse-tolerance recovery (acute repression with an incomplete rebound), persistent repression (acute and chronic suppression sustained), chronic-predominant repression (loss only under prolonged exposure on days 7,11), and classical tolerance (acute induction returning fully to baseline). Solid lines represent persistent and dynamic programs, while dashed black lines denote classical biphasic forward and reverse tolerance pathways.

The first analysis performed in this investigation was the phasic classification of all transcriptional changes according to distinct differentially expressed genes (DEG) dynamic patterns occurring at four time points (**Supplementary File 2**). These patterns are self-evident in the RNA-seq data from resting to acute (24 h) through to chronic inflammation (7 and 11 days), with only a subset conforming to the classical definition of tolerance as “diminishing returns,” or a system’s progressively weaker response to the same stimulus over time. Global patterns are summarized in **Figure 2**. Both arms converge at chronic, sustained DEG patterns from persistent bidirectional and or biphasic changes.

**Figure 3:**
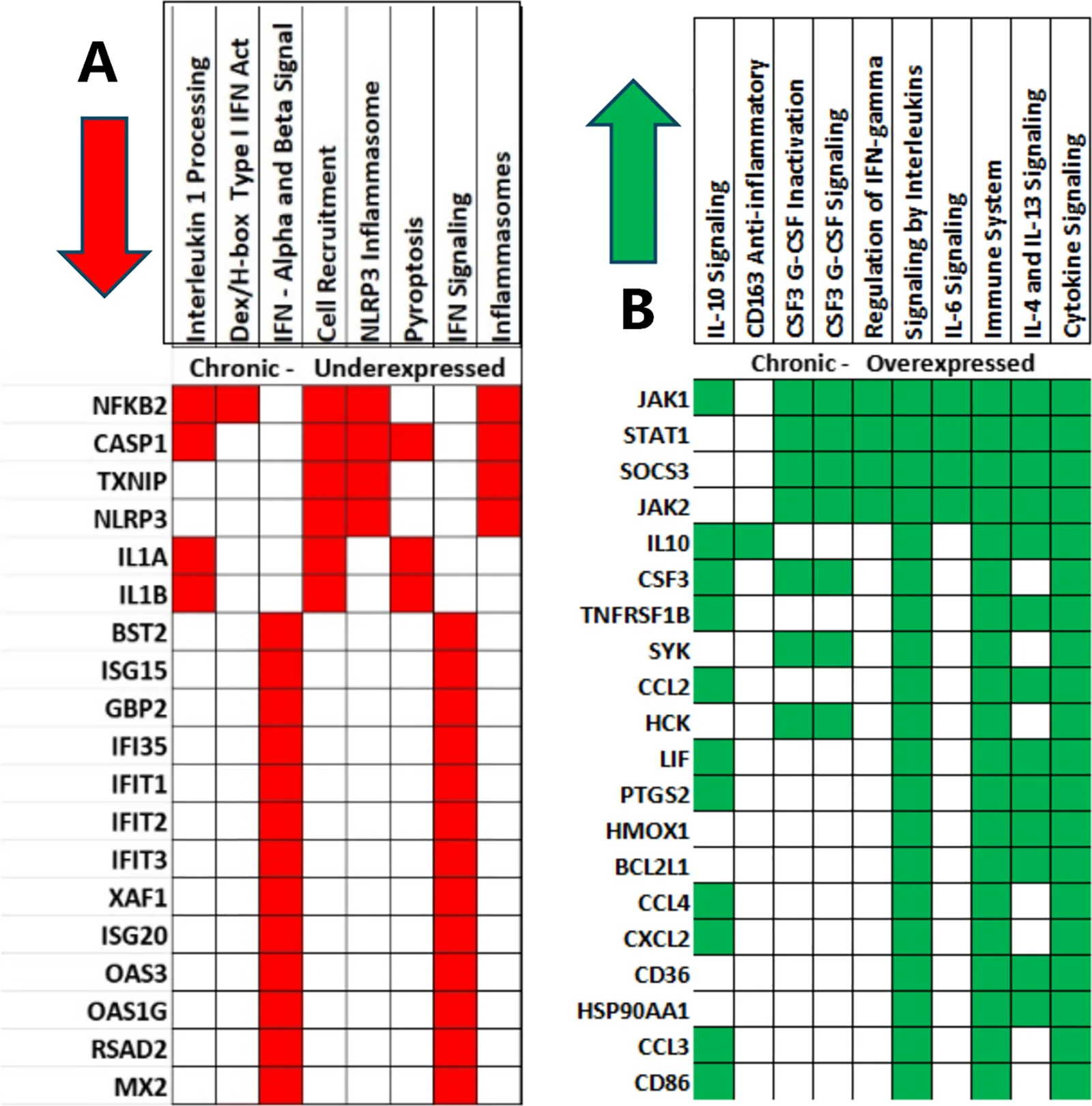
**(A)** Downregulated transcripts associated with chronic inflammation (7,11 days). Heatmap (red, left panel) shows the functional annotation of downregulated gene sets analyzed using Reactome bioinformatics, with genes on the Y-axis (left) and enriched pathways on the X-axis (top). Chronic inflammation was associated with marked transcriptional repression of type I IFN–associated pathways, pathogen-response–associated gene sets, IL-1α/IL-1β–related transcripts, inflammasome-associated genes, and antigen-presentation–associated pathways. Representative DEGs associated with tolerance-related transcriptional repression include (matched to panel): Ifi35 (–2.03 Log_2_FC, p = 1.4E–13); Rsad2 (–4.29, 9.2E–25); Casp1 (–0.93, 3.9E–07); Il1b (–4.62, 8.7E–22); Il1a (–5.29, 4.1E–08); Isg20 (–3.69, 2.0E–16); Oas3 (–2.63, 3.3E–68); Txnip (Reverse Tolerance Reduction vs baseline) (–0.90, 3.8E–04); Bst2 (–2.13, 1.7E–13); Nfkb2 (–2.08, 3.1E–12); Isg15 (–5.70, 3.7E–60); Nlrp3 (–3.15, 1.0E–118); Ifit1 (–6.45, 7.2E–25); Ifit3 (–5.11, 3.6E–44); Ifit2 (–2.78, 1.2E–05); Xaf1 (–3.83, 1.3E–66); Oas1g (–3.00, 2.2E–56); Gbp2 (–3.13, 4.0E–11); Mx2 (–4.52, 8.5E–93). Additional notable DEGs not represented in the heatmap during chronic inflammation included *Cd74*, *Fxyd2, Lsp1, Ifi27l2a*, and multiple MHC class I/II transcripts: *H2-Q6, H2-T24, H2-T23, H2-M2*, *H2-T10, H2-K2* and *H2-K1*. **(B)** Upregulated transcripts associated with chronic inflammation (7–11 days). The heatmap (green, right panel) displays the functional annotation of upregulated DEGs as defined by Reactome bioinformatics. Chronic inflammation was associated with strong upregulation of SOCS3/JAK–STAT signaling components, CD163-associated gene programs, and IL-10–related transcripts, CSF3/G-CSF and specific CCL chemokines. Representative DEGs (matched to panel) include: Socs3 (+4.73 Log_2_FC, p = 6.38E–125), Il10 (+7.38, p = 8.68E–82), Ccl2 (+5.18, p = 1.49E–22), Ccl4 (+1.80, p = 1.10E–16), Lif (+3.51, p = 4.12E–45), Tnfrsf1b (+2.74, p = 4.59E–222), Ptgs2 (+3.63, p = 8.50E–14), Csf3 (+3.32, p = 3.38E–03), Syk (+1.45, p = 2.99E–33), Jak2 (+1.43, p = 1.02E–07), Stat1 (+0.82, p = 9.08E–04), Cxcl2 (+3.02, p = 1.14E–13), Cd86 (+1.25, p = 1.86E–10), Hsp90aa1 (+1.89, p = 2.03E–10), Jak1 (+1.53, p = 1.14E–08), Hck (+0.89, p = 9.71E–10), Hmox1 (+1.13, p = 1.92E–13), Bcl2l1 (+1.51, p = 1.19E–11), Cd36 (+2.59, p = 3.80E–12), and Ccl3 (+1.35, p = 3.65E–10). Additional strongly upregulated DEGs not shown in the heatmap included *Klk9* (+7.2 Log_2_FC, *p* = 7.0E–10), *Nos2, Ccl7, Il1f9, S100a8, Csta3, Il33, Ccl12*, *Edn1, Sema3e, Urah*, *Vav1*, *Mast3*.

To identify major known pathways activated or repressed in established chronic tolerized macrophages versus acute and resting, the Reactome ontology platform generated heatmaps for underexpressed genes in **Figure 3A**, and chronically overexpressed genes in **Figure 3B**. These data demonstrate that, based on Reactome ontology, chronic inflammation is associated with the overexpression of IL-10–related transcripts, CD163-associated gene programs, SOCS/JAK–STAT signaling components, and CSF3, alongside the transcriptional repression of type I IFN–associated pathways and inflammasome-related gene sets.

To interpret the biological significance of differentially expressed genes in chronically tolerized macrophages, DAVID functional ontology analysis was performed using enriched biological processes, pathways, and molecular functions (**Tables 1A**, **1B**; truncated versions shown, with full results in **Supplementary File 1** and complete differential expression data in **Supplementary File 2**).

**Table 1A:** Enriched biological processes and pathways upregulated in chronic stimulation.

Database/database ID# ~ ontology	%	Sustained overexpressed in chronic	Fold enrich.	Benj. P-value
GOTERM_CC_DIRECT/GO:0005764~lysosome/GO:0033176~proton-transporting V-type ATPase complex	5.9	ABCD4, CALCRL, SRC, SPPL2A, TCIRG1, GNS, CLN5, GJA1, SNX1, CTSL, CTSK, PSAP, CAPN2, ACP5, LRRC8A, SLC15A3, SIAE, CTSD, CTSC, SNX6, SRGN, CD164, SLC31A2, VPS33A, SLC11A1, ATP6AP2, FUCA2, SLC11A2, GGH, MT1, MCOLN2, SLC39A14, HCK, LMBRD1, MARCKS, RNF183, ATP6V0C, SQSTM1/ATP6V0C, ATP6V1D, ATP6V1C1	2.7	1.74E-05
GOTERM_CC_DIRECT/GO:0001533~cornified envelope/GO:0030216~keratinocyte differentiation	1.9	SPRR2E, CSTA3, SPRR2F, SPRR2G, SERPINB2, SPRR2H, SPRR2I, SPRR2K, CSTDC4, SPRR2B, SPRR2D, STFA3/SPRR2E, CSTA3, SPRR2F, SPRR2G, SPRR2H, SPRR2I, SPRR2K, CFLAR, PTGS2, STFA3, CSTDC4, SPRR2B, SPRR2D	6.1	3.03E-04
INTERPRO/IPR000827:CC chemokine, conserved site/GO:0031726~CCR1 chemokine receptor binding/GO:0048247~lymphocyte chemotaxis/DOMAIN: Chemokine interleukin-8-like/KW-0145~Chemotaxis	1.1	CCL9, CCL12, CCL7, CCL6, CCL4, CCL3, CCL2/CCL9, CCL7, CCL6, CCL4, CCL3/CCL9, CCL12, CCL7, CCL6, CCL4, CCL3, CCL2, ADAM8//CCL9, CCL12, CCL7, CCL4, CCL3, CCL2, CXCL2/CCL9, CCL12, CCL7, CCL6, CCL4, C3AR1, CCL3, CCL2, FPR2, CXCL2, S100A8	14.8	4.37E-03
GOTERM_BP_DIRECT/GO:0071347~cellular response to interleukin 1/m_il22bpPathway: IL22 Soluble Receptor Signaling Pathway	2	CCL12, EDN1, SAA3, SERPINE1, CXCL2, CCL9, CCL7, CCL6, CCL4, ZC3H12A, CFL1, CCL3, CCL2/SOCS3, STAT1, JAK2, JAK1	5.3	2.18E-03
GOTERM_BP_DIRECT/GO:0007264~small GTPase mediated signal transduction/GO:0043547~positive regulation of GTPase activity/IPR003578:Small GTPase superfamily, Rho type	2.2	DOCK5, DOCK4, VAV1, RND1, RHOB, RAP1B, DOCK11, RHOT2, RASGEF1B, HMOX1, RAPGEF2, RHOV, RAPGEF5, NUCB2/CCL12, RABGAP1L, ARHGAP35, VAV1, DOCK11, CCL9, CCL7, RASGEF1B, CCL6, RGS1, CCL4, RACK1, CCL3, CCL2, RAPGEF2, RAPGEF5, MAPRE2, MAP4K4/RHOT2, RHOV, RND1, RHOB	4.3	4.98E-03
UP_SEQ_FEATURE/DOMAIN: Ig-like V-type/KW-0393~Immunoglobulin domain	2.2	CD86, CD274, CD84, CD83, CD80, CEACAM1, SLAMF9, SLAMF7, CD300E, SIGLEC1, MPZL1, CD200, NECTIN2, HAVCR2/CD86, CD274, CD84, CD83, CD80, SEMA3E, LILRB4A, LY9, FCGR1, MILR1, CEACAM1, ALCAM, SLAMF9, SLAMF7, CD300E, SIGLEC1, FCGR2B, MPZL1, CD200, NECTIN2, MFAP3L, HAVCR2	4.2	1.15E-02

Truncated (See **Supplementary File S1**). Columns report database/ontology ID and term, percent of annotated genes, gene list, fold enrichment, and Benjamini–Hochberg adjusted *p*-values. Dominant themes include higher expression of cytokine/cytokine-receptor signaling (chemokines CCL2/4/6/7/9/12; JAK–STAT: JAK1/2, STAT1/2, SOCS3), leukocyte chemotaxis (neutrophil/eosinophil/monocyte), FcγR-mediated phagocytosis, lysosomal/vesicular modules (V-ATPase, AP-3), cell–cell junction/adhesion, TNF/IL-1 responses, and NF-κB/MAPK regulators, small GTPase/Rho signaling, apoptosis/Bcl-2 family features, lipid handling (fatty-acid transport/sphingolipids), and ferroptosis components. This pattern is consistent with coordinated transcriptional enrichment associated with prolonged stimulation. Functional enrichment of DEGs increased during chronic stimulation, spanning GO (BP/CC/MF), KEGG, InterPro, SMART/UP features.

**Table 1B:** Enriched biological processes and pathways downregulated in chronic inflammation.

Database/database ID# ~ ontology	%	Sustained repression in chronic	Fold enrich.	Benj. P-value
GOTERM_BP_DIRECT/GO:0051607~defense response to virus	7.1	RTP4, SLFN8, CD40, IFITM2, UNC93B1, OAS1A, IFIT1, IFI44L, IFIT3, IFIT2, OAS1G, IFIH1, DHX58, CASP1, ITGAX, NLRP3, OASL1, OASL2, GBP3, ZBP1, APOBEC1, GBP5, GBP2B, IFI207, GBP7, RSAD2, IFI204, IFNGR1, MX2, BNIP3, TREX1, CARD9, ACOD1, ISG15, PARP9, ISG20, BST2, IL6, POLR3C, OAS3, IRF7, IFIT3B, IRF5, SHFL, MYD88, TLR2	6.8	1.45E-21
GOTERM_BP_DIRECT/GO:0032760~ tumor necrosis factor production/GO:0032640~tumor necrosis factor production	3.9	H2-T23, CSF1R, H2-T22, H2-Q6, PTAFR, H2-K1, H2-Q4, OAS1A, LPL, OAS1G, IFIH1, CLEC7A, LRRFIP2, CD14, IFNGR1, CARD9, CYBA, MIF, IL1A, IL6, TYROBP, OAS3, MYD88, H2-D1, TLR2/CARD9, AZI2, MYD88, TLR2	6.3	4.66E-10
GOTERM_BP_DIRECT/GO:0035457~cellular response to interferon-alpha/GO:0035458~cellular response to interferon-beta	1.2	IFI204, IFIT3B, OAS1A, IFIT1, GAS6, IFIT3, IFIT2, OAS1G/IFI209, GBP2B, IFI207, IFITM2, GBP7, IFI204, IFI205, IFI213, TREX1, IFI203, IFI211, IRGM1, OAS1A, ACOD1, IFI202B, IFIT1, IFIT3, OAS1G, MNDAL, IGTP, GBP2, GBP3/OAS3, TREX1, METTL3, OAS1A, ISG15, OAS1G	12.6	2.45E-04
KEGG_PATHWAY/mmu05134:Legionellosis	1.9	EEF1G, C3, CLK1, IL6, IL1B, BNIP3, CASP1, CD14, TNF, MYD88, NFKB2, TLR2	5.7	4.13E-04
GOTERM_BP_DIRECT/GO:0042832~defense response to protozoan	1.4	IL6, CD40, GBP2B, GBP7, IGTP, GBP2, MYD88, BATF, GBP3	7.2	2.71E-03
INTERPRO/IPR006117:2-5-oligoadenylate synthetase, conserved site	0.8	OAS3, OAS1A, OASL1, OAS1G, OASL2	23.2	4.75E-03
KEGG_PATHWAY/mmu05332:Graft-versus-host disease	1.7	H2-T24, H2-T23, IL1A, IL6, H2-T22, IL1B, H2-Q6, H2-K1, H2-Q4, TNF, H2-D1	5.2	2.04E-03
UP_SEQ_FEATURE/DOMAIN: Schlafen AlbA-2	0.8	SLFN5, SLFN8, SLFN2, SLFN1, SLFN4	21.8	7.31E-03

Truncated (See **Supplementary File S1**). The table reports the database and ontology ID, associated term, percentage of annotated genes, gene list, fold enrichment, and adjusted Benjamini–Hochberg p-values. Highlighted pathways include strong transcriptional repression of gene sets associated with antiviral response pathways, TNF-related signaling, type I interferon signaling, NOD signaling, antigen-presentation–associated pathways, and mitochondrial metabolic processes. Functional enrichment analysis of differentially expressed genes (DEGs) suppressed during chronic inflammation, performed using GO terms, KEGG pathways, and InterPro annotations.

**Figure 4:**
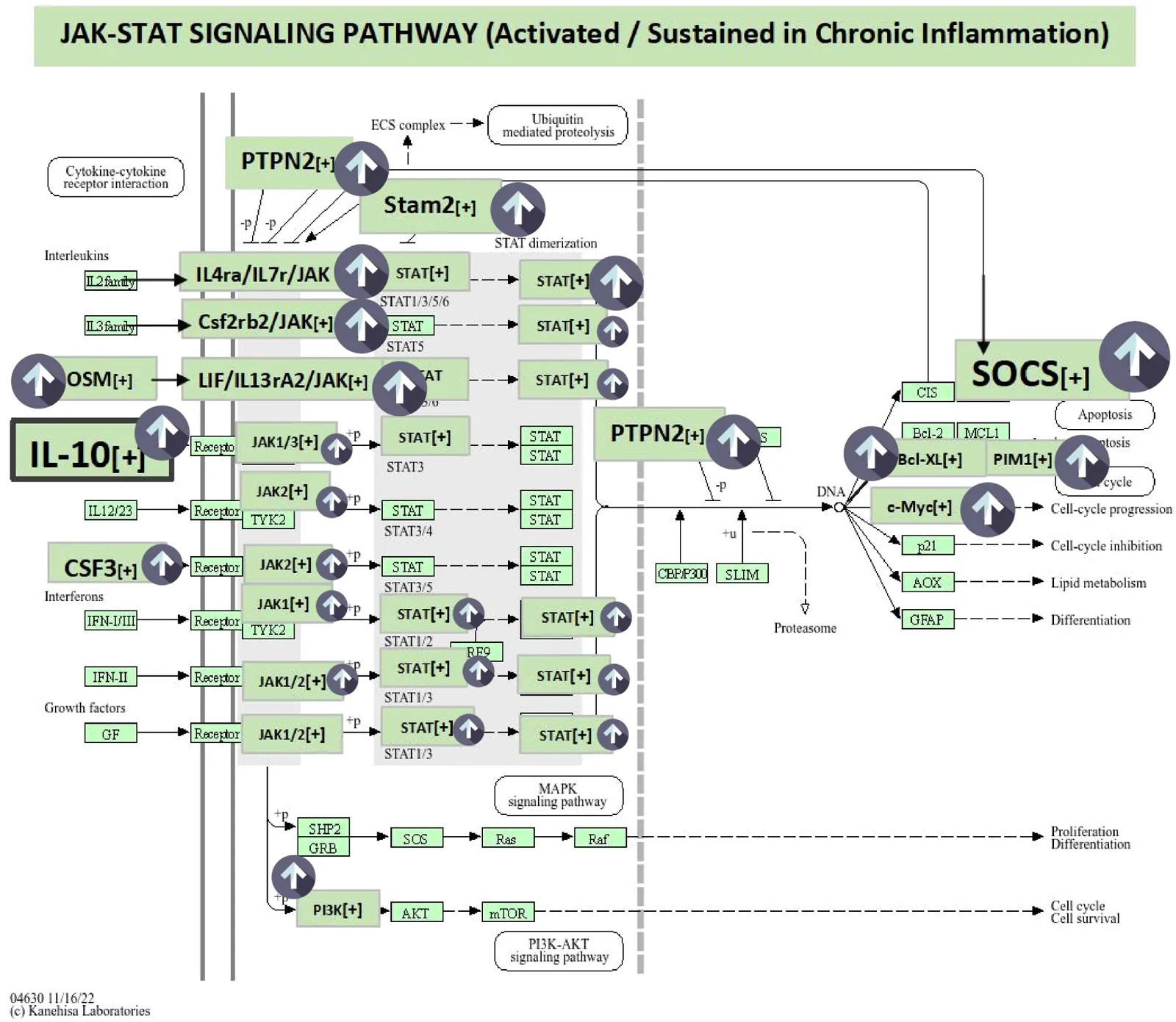
Upregulated Jak–STAT–associated gene expression under chronic stimulation. Differentially expressed genes (DEGs) upregulated in chronic inflammation were analyzed using DAVID pathway analysis, which highlighted the significant enrichment of the Jak–STAT/IL-10 signaling pathway. Pathway visualization highlights differentially expressed components(green overlays), including *Il10* (+7.3 Log_2_FC, *p* = 8.66E–82), *Socs3* (+4.7 Log_2_FC, *p* = 6.38E–125), *Lif* (+3.5 Log_2_FC, *p* = 4.12E–45), *Pim2* (+2.8 Log_2_FC, *p* = 7.31E–34), *Csf3* (+3.32 Log_2_FC, *p* = 3.37E–02), and *Ptpn2* (+1.28 Log_2_FC, *p* = 7.37E–09).

**Figure 5:**
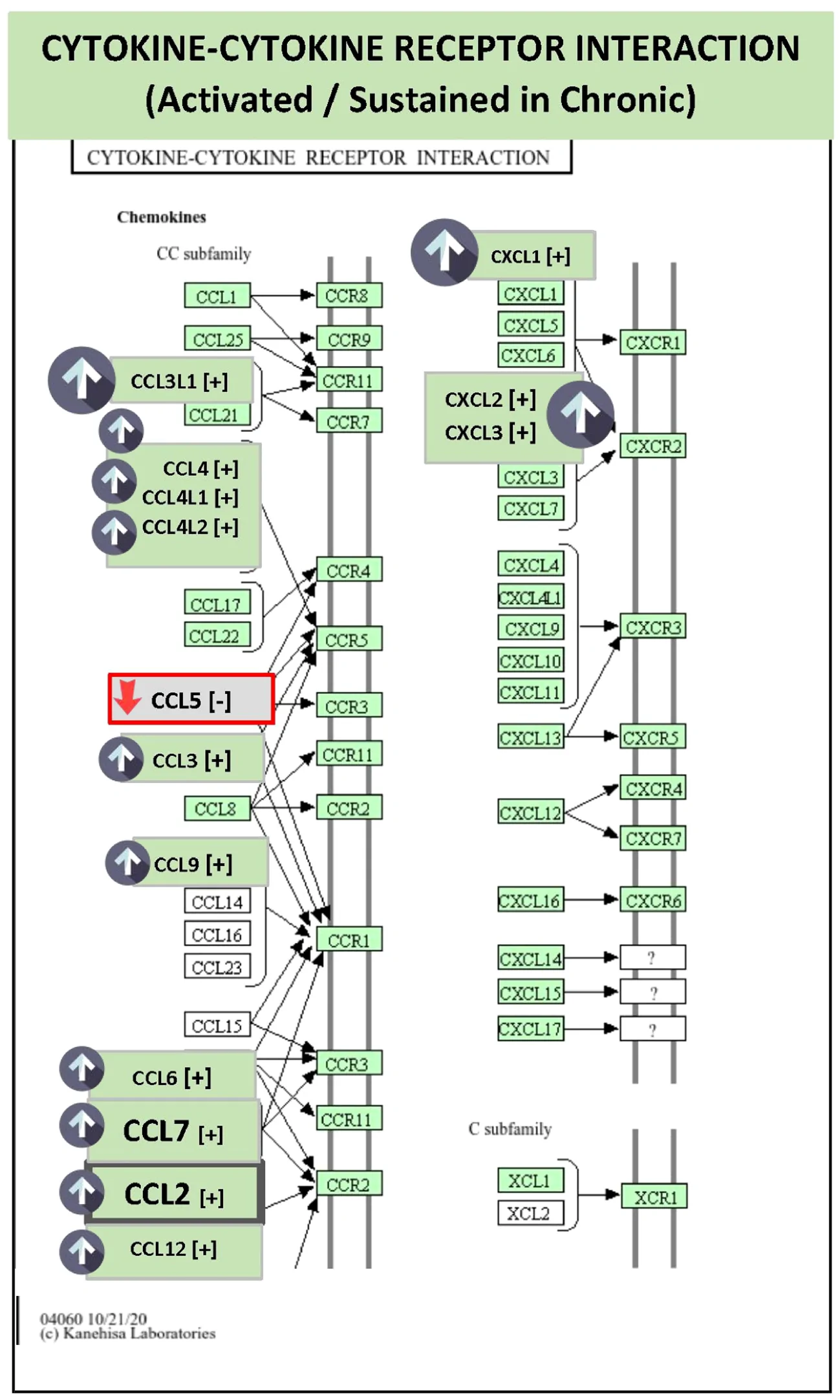
Enrichment of cytokine–cytokine receptor interaction–associated transcripts under chronic stimulation. DEGs upregulated in chronic inflammation were analyzed using DAVID pathway analysis, showing significant enrichment of the cytokine–cytokine receptor interaction pathway. Prominently upregulated transcripts included *Ccl7* (+5.64 Log_2_FC, *p* = 2.71E–21), *Ccl2* (+5.18 Log_2_FC, *p* = 1.49E–22), and *Ccl12* (+5.68 Log_2_FC, *p* = 2.17–E-17). Notably, a singular significant downregulation was observed for *Ccl5*.

**Figure 6:**
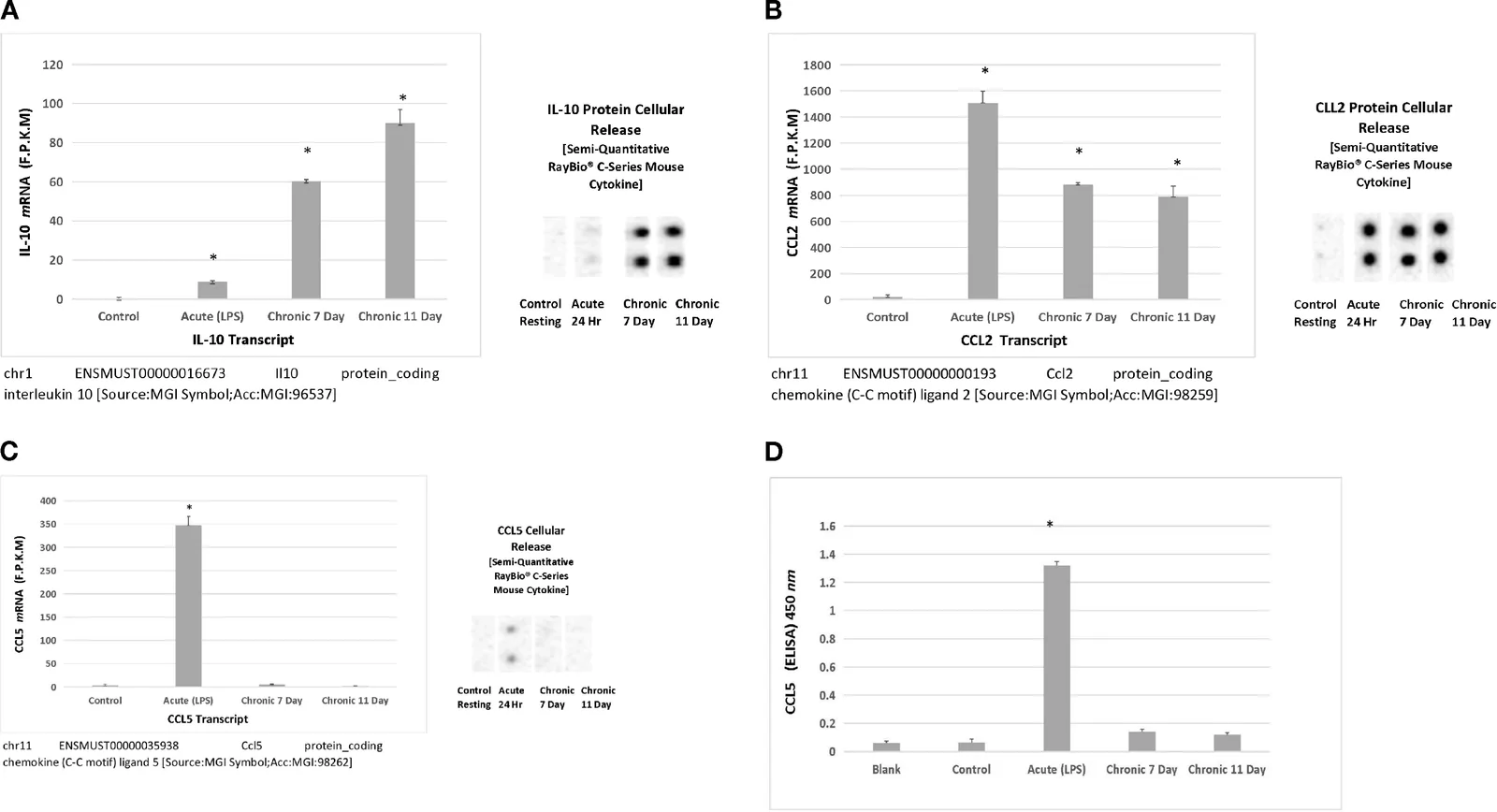
**(A)** IL-10 transcript and protein expression during chronic inflammation. IL-10 expression was assessed by RNA-seq (left) and semi-quantitative cytokine antibody array (right). Transcript levels (FPKM) increased significantly from acute (24 h) through chronic stimulation (7 and 11 days) compared with control (mean ± SEM, *n* = 3; *p* < 0.001). Protein release into the culture supernatant was confirmed by densitometry of chemiluminescent antibody array spots (RayBio C-Series), which were imaged using a Bio-Rad VersaDoc system. **(B)**. CCL2 transcript and protein expression during chronic inflammation. CCL2 expression was measured by RNA-seq (left) and a semi-quantitative cytokine antibody array (right). Transcript levels (FPKM) increased significantly during acute stimulation and remained elevated, though reduced, at 7 and 11 days of chronic stimulation compared with control (mean ± SEM, *n* = 3; *p* < 0.001). Protein release into the culture supernatant was confirmed by densitometry of chemiluminescent antibody array spots (RayBio C-Series), which were imaged using a Bio-Rad VersaDoc system. **(C)**. CCL5 transcript and protein expression during chronic inflammation. RNA-seq, cytokine antibody array, and ELISA were used to evaluate CCL5 expression. Transcript levels (FPKM) were significantly induced during acute stimulation but were suppressed under chronic conditions, consistent with a loss of responsiveness (tolerance). Protein release was confirmed by an antibody array (RayBio^®^ C-Series), with chemiluminescent densitometry images obtained using a Bio-Rad VersaDoc system. Data are expressed as mean ± SEM, *n* = 3; *p* < 0.001 compared with control. **(D)** ELISA validation of CCL5 protein release. Quantitative ELISA confirmed the acute induction of CCL5, followed by a severe loss of its secretion under chronic inflammation. Data represent absorbance at 450 nm (mean ± SEM, *n* = 3). Significance was determined via one-way ANOVA with Tukey *post hoc* test (*p* < 0.05).

Also, using DAVID (Database for Annotation, Visualization, and Integrated Discovery) Functional Annotation/Functional Ontology toolset, KEGG pathway maps were generated to provide greater specificity regarding signaling or associated genes in chronic inflammation (**Figure 4**: Jak/Stat–IL-10 signaling and **Figure 5**: cytokine–cytokine receptor interaction), with directional changes denoted by arrows. Among the most strongly upregulated cytokine/chemokine transcripts in chronic inflammation were IL-10 [+7.6 Log2FC] and Ccl2 [+5.2 Log2FC]. These genes were subsequently tested for transcript–protein correlations by spot testing (**Figures 6A–D**).

**Figure 7:**
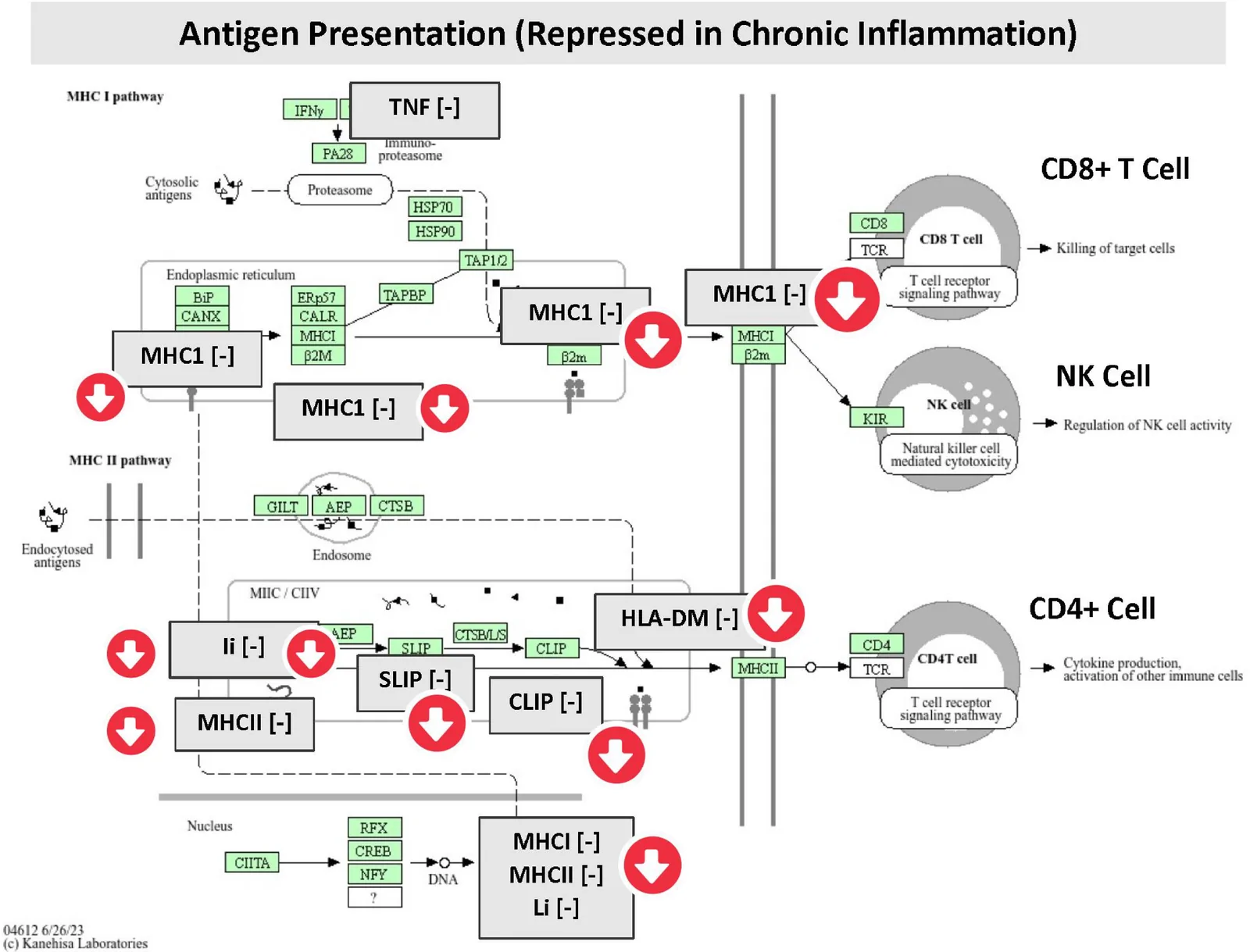
Transcriptional repression of antigen-presentation–associated gene sets under chronic stimulation. Differentially expressed genes (DEGs) under chronic stimulation were analyzed using DAVID pathway analysis, revealing transcriptional repression of gene sets associated with MHC class I and MHC class II pathways. Pathway mapping highlights reduced expression of multiple components (gray overlay boxes). CLIP denotes the class II–associated invariant chain peptide, a CD74-derived fragment that occupies the MHC-II groove prior to peptide loading by HLA-DM. Significantly downregulated transcripts, exhibiting either gradual reduction or classical tolerance dynamics, included MHC class I–associated genes H2-D1 (–1.54 Log_2_FC, p = 1.40E–08), H2-K1 (–1.90 Log_2_FC, p = 1.63E–14), H2-T23 (–1.94 Log_2_FC, p = 6.82E–19), H2-Q6 (–1.75 Log_2_FC, p = 5.57E–18), H2-Q5 (–2.63 Log_2_FC, p = 1.34E–06), H2-Q4 (–0.68 Log_2_FC, p = 3.55E–06), H2-T22 (–0.32 Log_2_FC, p = 4.02E–03), and H2-T24 (+0.68 Log_2_FC, p = 2.63E–01), as well as MHC class II–associated components including CD74 (–3.87 Log_2_FC, p = 7.84E–25) and H2-DMa (–0.96 Log_2_FC, p = 1.49E–02).

Transcriptional repression of antigen-presentation–associated gene sets are presented in **Figure 7** which highlights the loss of *H2-T23, H2-T22, H2-Q6, H2-K1, H2-Q4, PTPN22*, and *H2-D1*, along with dozens of transcripts essential for host type I IFN signaling (**Supplementary Table 1B**). Concomitant reductions included *ACOD1, GBP2, GBP2B, GBP3, GBP7, GAS6, IFI202B, IFI203, IFI204, IFI205, IFI207, IFI209, IFI211, IFI213, IFIT1, IFIT2, IFIT3, IFIT3B, IFITM2, IGTP, IRGM1, ISG15, METTL3, MNDAL, OAS1A, OAS1G, OAS3*, and *TREX1*.

Gene expression was quantified by quantitative real-time PCR (qPCR) and normalized to the housekeeping gene β-actin. Relative expression levels were calculated using the 2^-ΔΔCt method, with control (0 h) samples used as the baseline reference. Results are presented as log2 fold change relative to control. Statistical significance was determined using a two-tailed t-test on ΔCt values. Selected genes were chosen based on differential expression observed in the RNA-seq dataset to validate transcriptional changes during the indicated time points (**Table 2**).

**Table 2:** Quantitative PCR validation of selected genes identified by RNA-seq.

Gene	0 Hr	24 h log2FC	p-value	7 d log2FC	p-value	11 d log2FC	p-value
CD36	0	3.28	3.00E-03	4.09	1.00E-03	4.1	1.00E-03
CCL7	0	11.93	3.00E-04	7.37	2.00E-03	7.48	2.00E-03
NOS2	0	5.72	1.00E-03	5.29	1.00E-03	5.08	2.00E-03
IL1A	0	13.17	2.00E-04	5.02	3.00E-03	5.12	3.00E-03

Expression values are shown as log2 fold change relative to control, normalized to β-actin using the 2^-ΔΔCt method.

**Figure 8:**
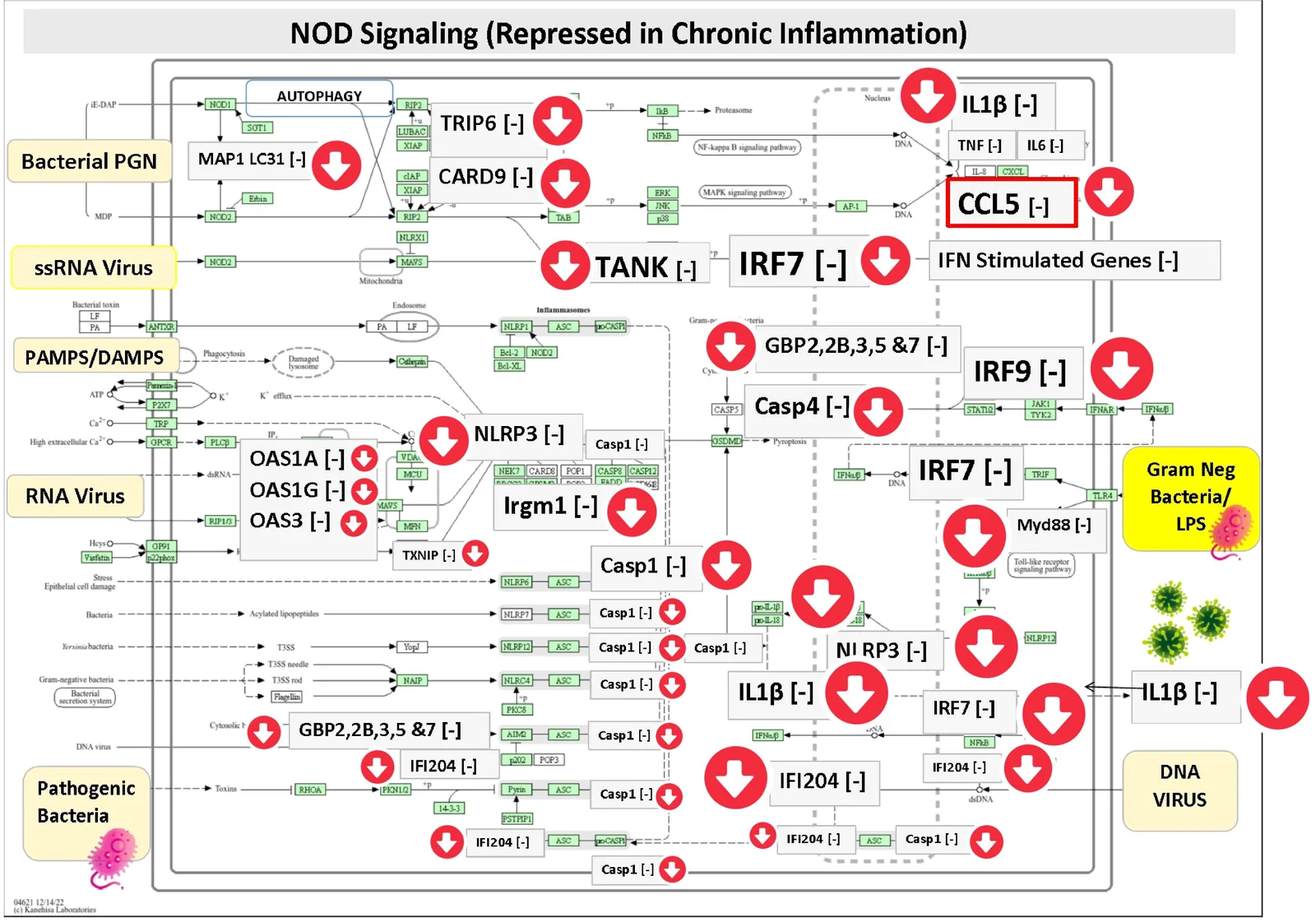
Transcriptional repression of NOD-associated gene sets under chronic stimulation. Differentially expressed genes (DEGs) repressed under chronic stimulation were analyzed using DAVID pathway analysis, revealing broad transcriptional repression of NOD-associated gene sets. Overlay boxes highlight downregulated components within the annotated pathway. Significantly reduced transcripts exhibiting either classical tolerance dynamics or gradual reduction during prolonged stimulation included genes annotated to NOD-related pattern-recognition and inflammasome-associated categories: Core sensors and inflammasome regulators: Nlrp3 (–3.15 Log_2_FC, p = 1.40E–118), Irf7 (–5.15 Log_2_FC, p < 1.00E–147), Irgm1 (–2.66 Log_2_FC, p = 1.14E–27), Tank (–0.73 Log_2_FC, p = 1.84E–02), Card9 (–0.34 Log_2_FC, p = 0.15), Trip6 (–0.92 Log_2_FC, p = 6.67E–04), Map1lc3a (–1.75 Log_2_FC, p = 2.56E–06). Caspases and effectors: Casp1 (–0.93 Log_2_FC, p = 3.93E–07), Casp4 (–1.61 Log_2_FC, p = 1.58E–29). OAS family members: Oas1a (–2.54 Log_2_FC, p = 1.04E–50), Oas1g (–3.00 Log_2_FC, p = 2.21E–56), Oasl1 (–4.21 Log_2_FC, p = 3.86E–123), Oasl2 (–6.13 Log_2_FC, p = 1.70E–87), Oas3 (–2.63 Log_2_FC, p = 3.27E–68).Guanylate-binding proteins (GBPs): Gbp2 (–3.13 Log_2_FC, p = 4.00E–11), Gbp2b (–3.23 Log_2_FC, p = 1.91E–23), Gbp3 (–2.39 Log_2_FC, p = 1.55E–13), Gbp5 (–2.75 Log_2_FC, p = 2.64E–03), Gbp7 (–1.91 Log_2_FC, p = 5.09E–14). Cytokines and downstream mediators: Il1b (–4.62 Log_2_FC, p = 8.70E–22), Il6 (–3.59 Log_2_FC, p = 5.53E–15), Tnf (–2.25 Log_2_FC, p = 1.49E–27). Interferon-related genes: Ifi44 (–3.55 Log_2_FC, p = 1.37E–24), Ifi203 (–2.38 Log_2_FC, p = 5.25E–29), Ifi204 (–2.62 Log_2_FC, p = 1.25E–21).Adaptor: Myd88 (–1.12 Log_2_FC, p = 2.10E–19).

In addition, as shown in **Figure 8**, chronic stimulation was associated with broad transcriptional repression of NOD-associated gene sets, alongside downregulation of transcripts annotated to pathogen-recognition and innate immune signaling pathways, including those linked to RNA virus–associated processes and pathways annotated for pathogen-associated molecular patterns (PAMPs), damage-associated molecular patterns (DAMPs), and microbial-associated molecular patterns (MAMPs) such as lipopolysaccharide, peptidoglycan, flagellin, and CpG DNA.

**Figure 9:**
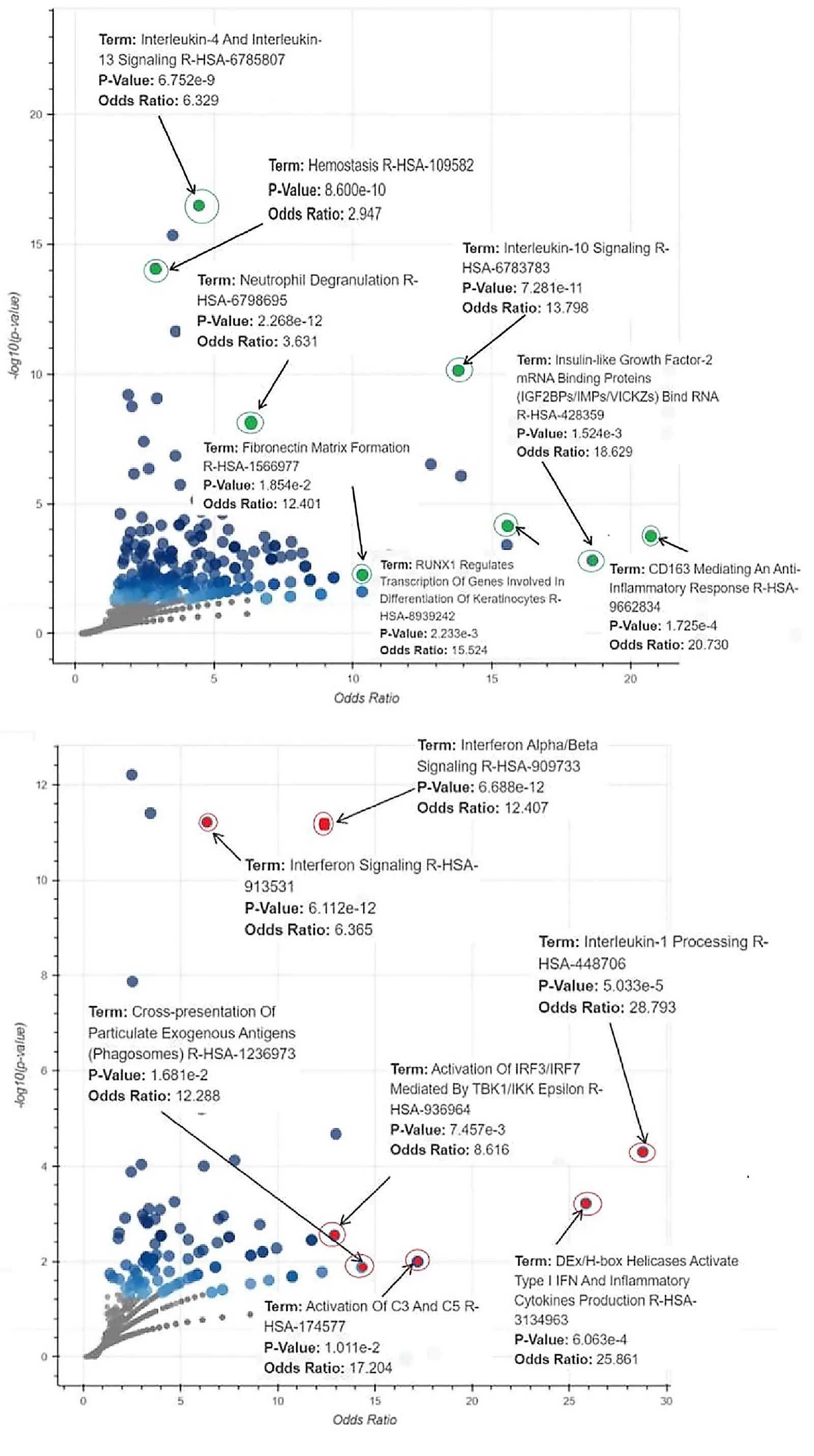
Reactome pathway enrichment associated with chronic stimulation.

A volcano plot generated from RNA-seq data analyzed using the Reactome bioinformatics platform revealed that chronic stimulation was associated with transcriptional enrichment of pathways shown in **Figure 9** (top panel) and transcriptional repression of pathways shown in **Figure 9** (bottom panel). Enriched pathway annotations included gene sets associated with neutrophil degranulation–related processes, IL-4 and IL-13–associated signaling, RUNX-related keratinocyte pathways, and IL-10–associated signaling. In contrast, repressed pathway annotations included antigen-presentation–associated gene sets, interferon-associated signaling pathways (with extensive repression), complement C3/C5–associated pathways, and cytokine-associated transcripts such as CCL5.

Volcano plots display Reactome-annotated pathway gene sets enriched under chronic stimulation based on RNA-seq data. The top panel shows transcriptionally enriched pathways, and the bottom panel shows transcriptionally repressed pathways. Each point represents a Reactome gene set; the x-axis indicates the odds ratio for enrichment, and the y-axis represents –log(p-value). Blue points denote significantly enriched pathways (p < 0.05), while gray points indicate non-significant terms. Highlighted points mark the most enriched annotations: green circles (top panel) indicate enriched pathway gene sets, and red circles (bottom panel) indicate repressed pathway gene sets. Representative annotated pathways include interleukin-associated signaling, interferon-associated signaling, complement-associated pathways, neutrophil degranulation–associated gene sets, and CD163-associated gene programs. Full pathway details are provided in **Supplementary File S1** (**Tables 1A**, downregulated; **Tables 1A**, upregulated).

**Figure 10:**
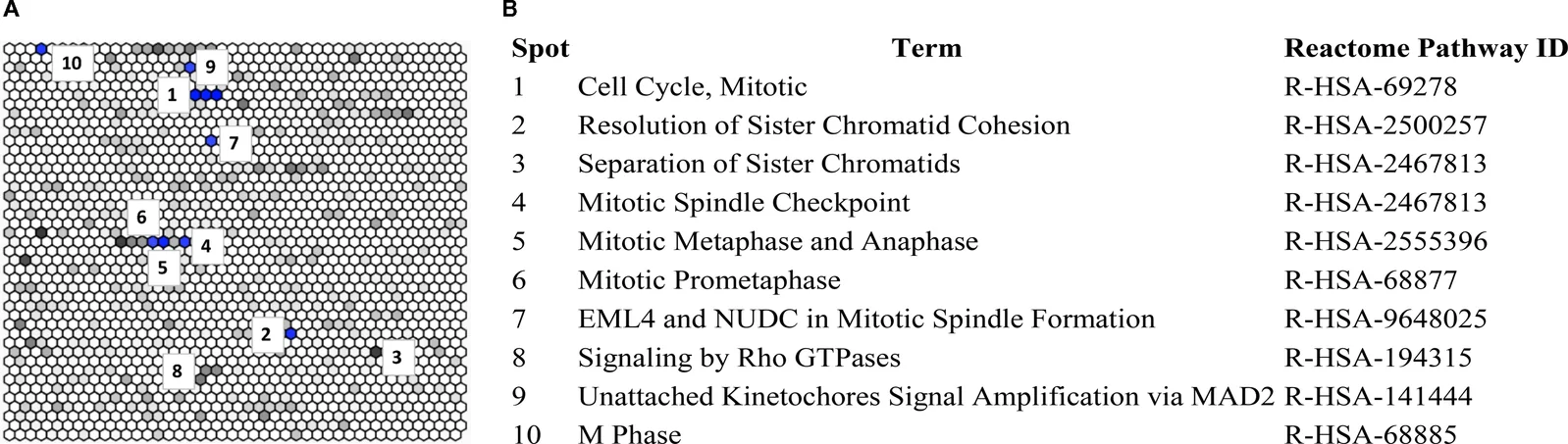
Reverse tolerance in chronic inflammation. **(A)** Hexagonal canvas plot of DEGs repressed during acute stimulation but restored to baseline under chronic conditions. Each hexagon represents a Reactome pathway term, with brighter intensity indicating greater Jaccard similarity between the input gene sets. Blue highlights mark the most significantly enriched pathways, labeled numerically for cross-reference. **(B)** Tabulated interpretation of Reactome pathways corresponding to numbered spots in panel **(A)** Listed are the pathway terms, Reactome IDs, and their association with reverse tolerance.

We discuss the category of reverse tolerance separately (**Figure 10**), rarely discussed in the literature, using a hexagonal canvas plot that is defined by transcripts repressed during acute inflammation and those that return to baseline during chronic conditions. Most of these are linked to regulated homeostatic processes, particularly cell cycle and mitotic pathways, which normalize in the chronic state. While these results are broad, they lay the groundwork for a more comprehensive interpretation of the dataset, which is further discussed in the following section.

## Discussion

Macrophages are primary sensors of microbial signals and central regulators of the transition from activation to immune-regulatory states, propagating feedback through multicellular networks that define chronic inflammation and the development of tolerance (41, 42). It is imperative that tolerance is studied in detail, as it plays a central role in immune regulation, particularly in contexts such as tumor-associated loss of immune surveillance described in the literature. In this study, we examine the transition from resting through acute to chronic stimulation in RAW 264.7 cells, which resulted in bidirectional differential gene expression (DEG) responses clustered into eight transcriptionally distinguishable patterns—many of which did not align with classical definitions of tolerance or reverse tolerance. In the following sections, we highlight changes that converged on either sustained overexpression or stable underexpression at days 7 and 11.

### Overexpressed in acute and sustained through chronic

**Figure 11:**
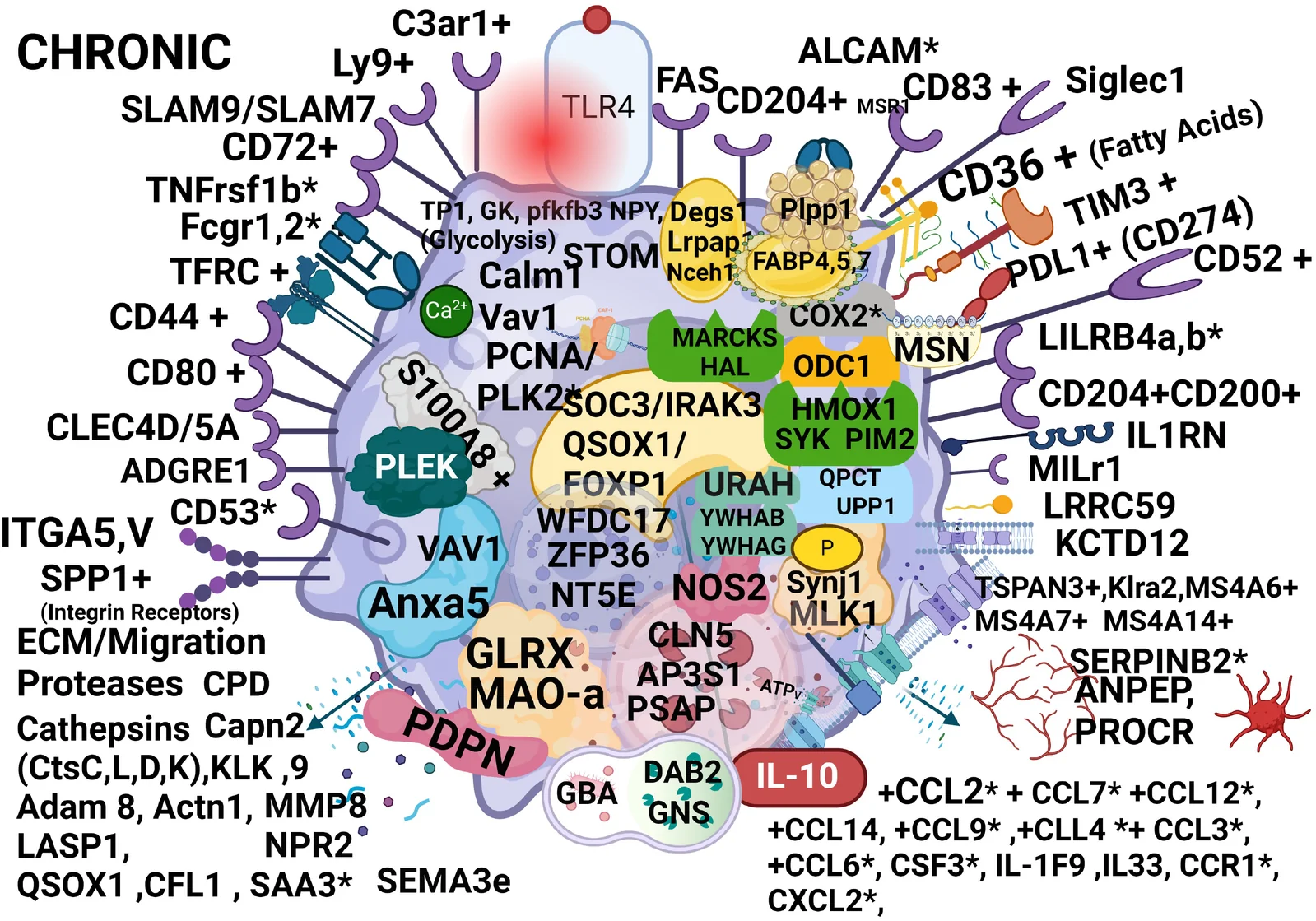
Sustained transcriptional induction of gene programs under chronic stimulation. The schematic depicts transcripts that are upregulated during acute stimulation and remain elevated under chronic conditions. Categories include surface glycoproteins, integrins, scavenger receptors, intracellular regulators, metabolic enzymes, proteases, and signaling intermediates. Immune checkpoint–associated transcripts such as PD-L1 (CD274) and TIM-3 (HAVCR2) are shown within a broader network of persistently induced genes, including SPP1 (osteopontin), MARCKS, CD204 (MSR1), LILRB4, and S100A8. Genes marked with [*] represent tolerance-linked transcriptional dynamics, characterized by peak expression during the acute phase followed by partial decline while remaining above baseline during chronic stimulation. Collectively, these pathways illustrate coordinated transcriptional programs associated with prolonged inflammatory signaling, including extracellular matrix remodeling, metabolic adaptation, and receptor-associated signaling.

Many genes were elevated during the acute phase and remained elevated throughout chronic stimulation, suggesting persistent transcriptional induction rather than classical tolerance. These included immune checkpoint–associated transcripts such as CD274 (PD-L1) and its scaffold protein moesin (MSN), a component of the ezrin–radixin–moesin (ERM) complex (43, 44) as well as HAVCR2 (TIM-3) (**Figure 11**). In other experimental systems, blockade of PD-L1 or TIM-3 has been shown to enhance CD8^+^ T cell activity and reduce regulatory T cell–associated suppression across multiple cancer models (45, 46) (**Figure 11**); however, functional immune consequences were not directly assessed in the present study.

#### Integrins

Sustained transcriptional overexpression of extracellular matrix (ECM)–associated components was observed under chronic stimulation, including MARCKS (47, 48) and SPP1 (osteopontin). In other experimental systems, these genes have been reported to be co-expressed with immune checkpoint molecules such as PD-L1 and CTLA-4 and associated with the recruitment of dysfunctional T cells and NK cells, as well as resistance to immunotherapies in aggressive cancers (49–59). Therapeutic strategies targeting these molecules—including monoclonal antibodies such as hu1A12, OPN-BsAb, and AOM1—are currently under investigation (60, 61) and related approaches have been explored in contexts involving M2-polarized macrophages (62). Additional integrin-related transcripts persistently upregulated under chronic stimulation included ITGA5 and macrophage scavenger receptor 1 (MSR1/CD204), both of which have been described as candidates for antibody-based therapeutic targeting in the literature (63–67) See review (68). Elevated expression of leukocyte cell adhesion molecule (ALCAM) was also observed a molecule that has been explored as a target for antibody–drug conjugates such as praluzatamab ravtansine (CX-2009), currently in phase II clinical trials. Notably, functional immune or therapeutic effects were not directly assessed in the present study.

#### Checkpoints

In addition to PD-L1, the data revealed sustained transcriptional overexpression of several immune checkpoint–associated genes previously described in the literature, including C3AR1, a complement receptor implicated in immune-regulatory contexts (69–75), and human leukocyte immunoglobulin-like receptors (LILRs), specifically LILRB4A and LILRB4B, which have been reported in M2-like tumor-associated macrophages (76). In other experimental systems, these molecules have been associated with expansion of myeloid-derived suppressor cells (MDSCs) and altered activity of NK, T, and B cell populations (77–80); however, such functional immune effects were not directly evaluated in the present study. Notably, members of the LILRB receptor family have also been described as mediators of fetal tolerance at the maternal–fetal interface, where they contribute to modulation of NK cell activity and immunological adaptation to fetal antigens—mechanisms that have been proposed to share conceptual parallels with tumor-associated antigen tolerance in the literature (80, 81).

Overall, the transcriptional patterns observed in this study align with literature-reported immune-regulatory programs, including coordinated overexpression of LILRB receptors together with elevated IL-10, CD83 (82), and TNFRSF1B (TNFR2) (83), all of which have been described in association with non-responsiveness to immune checkpoint inhibitor therapy in other experimental and clinical contexts (84). Collectively, the transcriptional profile identified here resembles immune-regulatory states reported in models of chronic inflammation and sepsis, characterized by broad repression of antigen-presentation– and interferon-associated gene programs (85–90). Importantly, functional immune impairment or therapeutic non-responsiveness was not directly assessed in the present study.

In this category, we also identified chronically overexpressed checkpoint-associated transcripts that have been investigated as therapeutic targets in other experimental and clinical settings, particularly in efforts to modulate T-cell receptor signaling and interferon-associated pathways. These included CD73 (NT5E) (91, 92), C3AR1 (93, 94), CD204 (68), CD200 CD53+ (95–100), CD44 (101, 102), Fcgr1 Fc receptor, IgG, high affinity (CD64(+)) (103), SLAMF9 (CD84-H1; SF2001; CD2F10) (104), and SLAMF7(+) (CRACC; CS1; CD319) (105). In other disease contexts, targeting some of these molecules—such as SLAMF7 with monoclonal antibodies including elotuzumab—has been shown to modulate antibody-dependent cellular cytotoxicity and NK cell activity, including in multiple myeloma (106). Functional immune effects were not directly evaluated in the present study (106).

#### Signaling systems

Transcriptional patterns observed during the chronic phases of this study align with gene programs reported in tumor-associated macrophage (TAM) and M2-like macrophage states in the literature, including elevated expression of suppressor of cytokine signaling 3 (SOCS3), sustained induction of JAK/STAT– and IL-10–associated transcripts (107–109), and increased expression of the serine/threonine protein kinase Pim-2. In other experimental systems, these pathways have been implicated in immune-regulatory macrophage states and tumor-associated immune evasion (110–112); however, functional signaling activity and tumor immune outcomes were not directly assessed in the present study.

#### Extracellular matrix calcium binding proteins/cathepsins, proteases

Data from the chronic phases of this study also revealed sustained transcriptional overexpression of S100A8, a calcium-binding protein that has been reported in the literature to be involved in neutrophil-associated inflammatory responses and is frequently associated with inflammatory pathologies such as COVID-19, septic shock, and cancer, where it is often co-expressed with PD-L1 (113–117). In other experimental contexts, S100A8-associated gene programs such as ADAM 8 have been linked to extracellular matrix remodeling and altered cell motility (118); however, functional immune or migratory processes were not directly assessed in the present study.

Data from the chronic phases of this study also revealed sustained transcriptional overexpression of multiple protease- and matrix-associated genes previously described in tolerized macrophages, chronic inflammation, and cancer, including cathepsins C, D, and L, ADAM8, carboxypeptidase D (CPD), and kallikrein-related peptidase KLK9, alongside reduced expression of the endogenous protease inhibitor cystatin C (CST3) (119–123).

Additional persistently induced transcripts included tetraspanins such as TSPAN3, which have been reported in the literature to participate in microbial interactions (124, 125), cell adhesion, and migration (126) and have been associated with inflammatory and tumor immune escape (127–133). Quiescin sulfhydryl oxidase 1 (QSOX1), a gene previously linked to extracellular matrix remodeling and tumor invasion and currently under investigation as a therapeutic target in breast cancer models, was also transcriptionally elevated (134–136). Podoplanin (PDPN), which has been described in other experimental systems in association with epithelial–mesenchymal transition, immune-regulatory tumor microenvironments, and resistance to targeted therapies, was similarly overexpressed (137–142). Finally, increased expression of PLAUR (CD87), the urokinase plasminogen activator receptor, was observed; this molecule has been extensively reported in the literature as associated with matrix remodeling, cell migration, and aggressive tumor progression (143, 144).

#### Chemokines

Data from the chronic phases of this study revealed sustained transcriptional overexpression of several chemokines previously described in chronic inflammation and tumor-associated contexts, including CCL2 (118) and CCL7. In other experimental systems, these chemokines have been reported to be associated with myeloid-derived suppressor cell (MDSC)–rich tumor microenvironments and resistance to immunotherapies (145, 146), as well as with aggressive cancers and poor clinical outcomes (147–149). Additional chemokines transcriptionally elevated under chronic stimulation included CCL12 and CXCL2, which have been described as components of the CCL2/CCL12–CCR2 axis implicated in immune cell trafficking within tumors in the literature (42). These pathways have also been explored as therapeutic targets, including through monoclonal antibody–based strategies aimed at enhancing anticancer vaccine efficacy in other contexts (150). Overexpression of QPCT was likewise observed; this enzyme has been reported to modulate CCL2/CCR2-associated chemokine activity under chronic inflammatory conditions (151). Functional immune recruitment, therapeutic responses, or clinical outcomes were not directly assessed in the present study.

#### Growth factors; chemokines

Data from this study revealed persistent transcriptional elevation of granulocyte colony-stimulating factors (CSF3/G-CSF) during chronic stimulation, remaining above baseline while reduced relative to acute induction, consistent with partial tolerance dynamics. In the literature, G-CSF has been described as a key regulator of neutrophil mobilization and is widely used therapeutically as a recombinant protein for the treatment of neutropenia, chemotherapy-induced myelosuppression, and invasive fungal infections (152). Among chemokines, the most prominent transcript exhibiting classical tolerance dynamics during chronic stimulation was CCL5, which has been reported in other systems to participate in the CCL5/CCR5 axis and to influence dendritic cell and CD8^+^ T-cell–associated immune responses, although findings have not been fully consistent across studies (153–155).

Notably, prior studies have reported that tumors expressing high levels of G-CSF are associated with poor clinical prognosis, particularly in high-grade malignancies (156, 157) and have linked G-CSF–rich tumor microenvironments to expansion of immunoregulatory neutrophil populations, angiogenesis, and stromal remodeling (41). In contrast, recombinant G-CSF is clinically regarded as safe and effective for reversing therapy-induced neutropenia (158–160). Preclinical models have further suggested that G-CSF can influence tumor growth, vascularization, and the expansion of immune-regulatory and endothelial progenitor cell populations in tumor-bearing mice (161). Together, these reports highlight the context-dependent and potentially dual roles of G-CSF in cancer biology. Importantly, functional immune effects, tumor outcomes, and therapeutic responses were not directly evaluated in the present study.

#### Metabolic rewire

Among the most prominent transcriptional changes observed under chronic stimulation was sustained overexpression of CD36, a fatty acid translocase that has been reported in the literature to be induced downstream of Toll-like receptor signaling (e.g., TLR4, TLR6, TLR7) and to feature prominently in tumor-associated macrophage and M2-like macrophage gene expression signatures (162, 163). In other experimental systems, CD36-associated transcriptional programs have been linked to altered lipid handling, immune-regulatory tumor microenvironments, and unfavorable clinical outcomes, where CD36 frequently emerges as a hub gene in systems-level analyses (164–169).

Elevated expression of spleen tyrosine kinase (SYK) was also observed and has been reported in prior studies in association with lipid accumulation and metabolic disorders such as atherosclerosis (168, 169). Additional metabolic-related transcriptional changes included altered expression of fatty acid–binding protein (FABP) family members, including Fabp7, Fabp5, and Fabp4, which have been described in the literature as components of lipid storage and metabolic adaptation programs in macrophages and other cell types (170, 171). Overexpression of Ptgs2 (COX2) was likewise observed, consistent with prior reports linking lipid-associated transcriptional programs to arachidonic acid–related pathways and prostaglandin-associated signaling in inflammatory and tumor-associated contexts) (162, 163, 172, 173). Importantly, metabolic flux, immune suppression, or tumor-promoting activities were not directly assessed in the present study.

Bidirectional metabolic transcriptional changes observed under chronic stimulation included reduced expression of plasma phospholipid transfer protein (PLTP), a lipid transfer and lipopolysaccharide-binding protein that has been reported in the literature to be associated with altered immune polarization and cholesterol handling (174, 175). Widespread transcriptional repression of oxidative phosphorylation (OXPHOS)–associated genes was also observed, including mitochondrial-encoded transcripts (mt-Nd4, mt-Nd6, mt-Te, mt-Cytb), ATP synthase components (Atp5e, Atp5g2), the complex III–IV supercomplex assembly factor Higd2a (176, 177),and NDUFS7, a core subunit of NADH:ubiquinone oxidoreductase, consistent with OXPHOS-related gene expression patterns reported in immune-regulatory and M2-like macrophage states (178).

Additional repressed transcripts included Atpif1, CARD19, and Cmpk2, which have been described in other systems as contributors to mitochondrial structure, homeostasis, and innate immune signaling (179–182). Similar mitochondrial transcriptional perturbations have been reported in models of inflammation-associated metabolic remodeling, including acute inflammation–induced OXPHOS alterations (62, 183). Notably, reduced expression of immune-responsive gene 1 (IRG1/ACOD1) was also observed. ACOD1 has been reported in the literature to encode a mitochondrial enzyme linking immune metabolism to inflammatory responses through itaconate production, with dysfunction described in a range of inflammatory and metabolic disorders (184–189). In contrast its end product may have therapeutic potential as itaconate derivatives including dimethyl itaconate (DI), 4-octyl itaconate (4-OI), and 4-ethyl itaconate (4-EI) have been explored for potential roles in cancer biology and immunotherapy (190–192). In cancer biology, higher expression of ACOD1 has been reported to exert antitumor effects by enabling higher itaconate production, which can enhance the efficacy of immune checkpoint blockade (anti-PD-1/PD-L1 therapy) (193).

One of the most unexpectedly elevated differentially expressed genes under chronic stimulation was Nos2, accompanied by marked transcriptional reduction of Sod2, and concomitant elevation in COX2, a condition leading to elevated accumulation of O2 and NO and ONOO nitrosative stress, which could exacerbate mitochondrial dysfunction, consistent with rapid glycolytic shift associated with immune tolerance (194, 195). Our findings align with emerging evidence that NOS2/COX2 elevation in the TME is associated with a clinical signature of immunosuppression associated with ER estrogen receptor-negative (ER-) breast cancer, linked to metastasis, drug resistance, cancer stemness, and immune suppression (196). Moreover, in the present study, persistent Nos2 overexpression was accompanied by increased expression of transcripts previously associated with nitrosative or oxidative stress–related responses in the literature, including Glrx, and Hmox1 (197, 198), S100A8 (199), QSOX1 (200, 201), glutaredoxin (Glrx), and heme oxygenase (Hmox1) (202–204).

#### Iron metabolism

Chronic stimulation in this study was associated with sustained transcriptional overexpression of transferrin receptor 1 (Tfrc) alongside reduced expression of multiple genes involved in intracellular iron–sulfur cluster assembly and iron handling, including HscB, Iscu, Cisd3, and the ferritin heavy chain Fth1. Similar transcriptional patterns have been reported in the literature in association with M2-like and tumor-associated macrophage states, where altered iron-handling gene programs have been linked to immune-regulatory microenvironments (205). In other experimental systems, dysregulated macrophage iron metabolism has been associated with tumor-associated immune evasion and regulatory T-cell–rich tumor microenvironments (191); however, functional iron storage capacity, immune escape, or T-cell infiltration were not directly assessed in the present study.

#### Novel signature checkpoint genes

One of the most striking transcriptional features observed under chronic stimulation in this study was sustained overexpression of a family of small proline-rich proteins (SPRRs), including SPRR2B, SPRR2D, SPRR2E, SPRR2F, SPRR2G, SPRR2H, SPRR2I, SPRR2J, and SPRR2K, which, to our knowledge, has not been previously reported in macrophages. These transcripts represented among the largest-magnitude transcriptional changes detected, with multiple family members exceeding +7 Log_2_ fold change. Despite their magnitude, relatively little is known about the functional biology of SPRRs outside their established roles in the cutaneous barrier. In other experimental systems, SPRRs have been reported to be induced downstream of TLR4/MYD88 signaling and to exhibit bactericidal activity against methicillin-resistant Staphylococcus aureus (MRSA) (206). SPRR family members have also been linked to chronic inflammatory diseases (207, 208), and to responses to noxious stimuli such as cigarette smoke (21). The functional significance of SPRR induction in chronically stimulated macrophages remains to be determined, however recent evidence supports a role in EMT, the engagement in p53 signaling and in protection from DNA damage (209).

### Downregulated in acute and repressed in chronic stimulation

**Figure 12:**
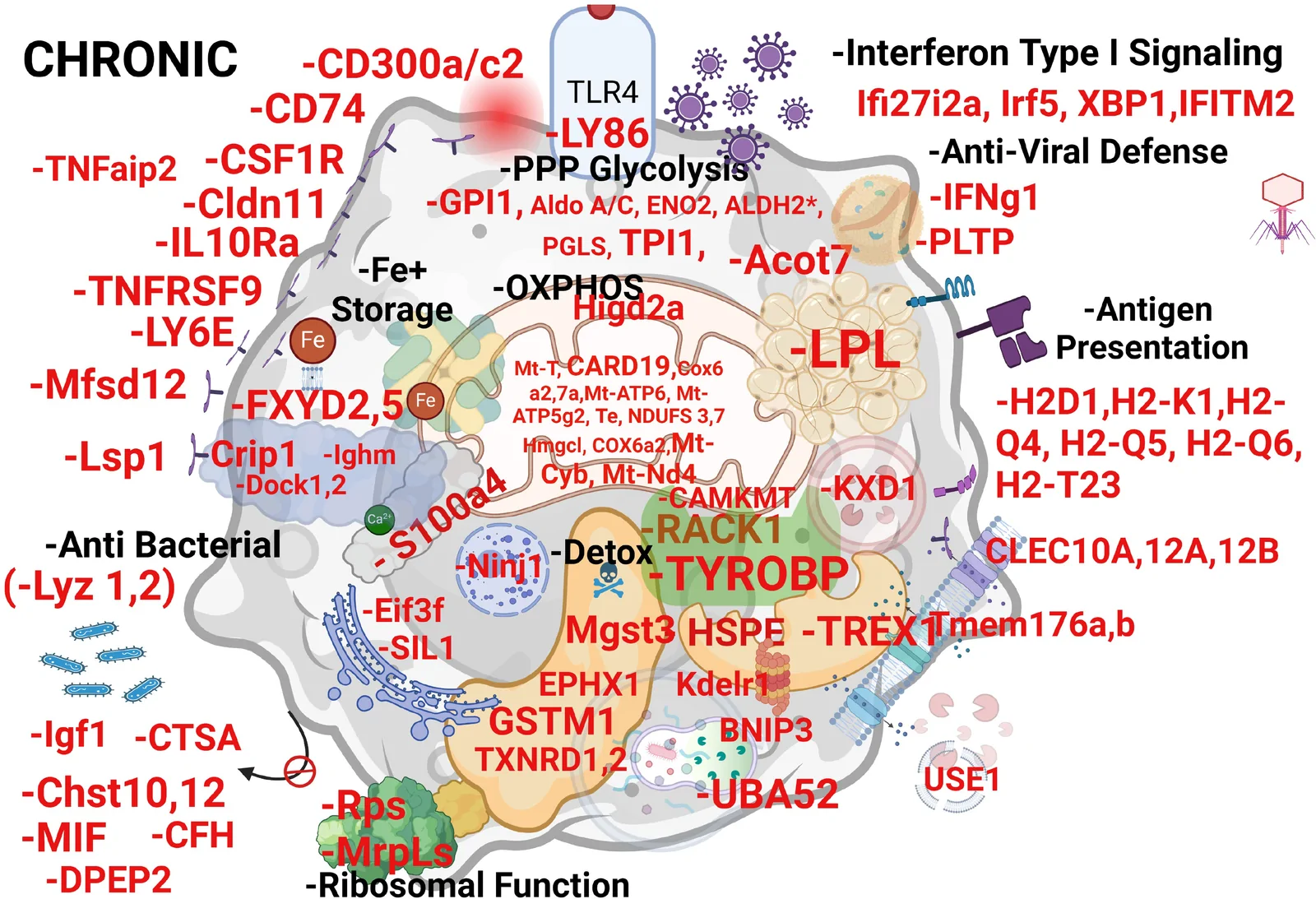
Transcriptional repression of gene programs under chronic stimulation. The schematic highlights transcripts that are acutely suppressed during LPS stimulation and remain repressed at chronic endpoints. Persistently reduced expression was observed across multiple gene programs annotated to immune-related and metabolic pathways, including MHC class I–associated transcripts (H2-D1, H2-K1, H2-Q4, H2-Q5, H2-Q6, H2-T23), type I interferon–associated gene families (e.g., IFIT, IFI, OAS, SLFN), antibacterial-associated transcripts such as lysozymes (Lyz1, Lyz2), oxidative phosphorylation (OXPHOS), glycolysis, iron-handling genes, detoxification pathways, and ribosomal components. Additional repressed transcripts (**Figure 13**) included metabolic regulators such as ACOD1, pattern-recognition–associated genes (e.g., CD14, CLEC4E), and acute-phase–associated genes such as SAA3. Collectively, these patterns illustrate coordinated transcriptional repression of multiple immune- and metabolism-associated gene sets during prolonged stimulation.

Under expressed DEGs exhibited sustained repression from acute through chronic stimulation, including multiple gene programs previously associated with immune regulatory states, as shown in **Figure 12**.

#### Antigen presentation

One of the most pronounced transcriptional changes observed under chronic stimulation was sustained repression of multiple histocompatibility-related genes, including H2-Q4, H2-Q6, H2-T23, H2-D1, H2-T22, H2-T24, H2-K1, and H2-Q5. These genes are annotated to antigen-presentation pathways and have been extensively described in the literature as components of host immune recognition processes. Similar transcriptional repression of MHC class I–associated genes has been reported in models of chronic inflammation, infection, and cancer, where reduced MHC expression has been linked to altered immune microenvironments (208). For example, Xiong et al. demonstrated that the loss of H2-K1 and H2-D1, together with impaired interferon signaling, was associated with reduced CD8^+^ T-cell infiltration and enhanced tumor progression in murine models (209). In line with these prior studies, concurrent repression of MHC class I–associated transcripts and type I interferon–related gene programs observed here may reflect immune-regulatory transcriptional states reported in chronic inflammatory contexts (210, 211). For example, Xiong et al. demonstrated that loss of H2-K1 and H2-D1, together with impaired interferon signaling, was associated with reduced CD8^+^ T-cell infiltration and enhanced tumor progression in murine models (210). In other biological systems, modulation of nonclassical MHC genes such as H2-Q5 has been described at the maternal–fetal interface to limit immune activation (212), and therapeutic strategies targeting antigen-presentation pathways—including CD40 agonists—have been explored to enhance immune activation in cancer models (213, 214). Functional antigen presentation, immune surveillance, vaccine responsiveness, or therapeutic effects were not directly evaluated in the present study.

#### Interferon-associated gene programs

Transcriptomic analysis revealed broad and sustained repression of interferon (IFN)–associated gene programs under chronic stimulation, encompassing multiple interferon-inducible genes (IFIs), interferon-induced proteins with tetratricopeptide repeats (IFITs), interferon regulatory factors (IRFs), interferon receptors (IFNGR1), interferon-induced helicases (IFIH1), 2′–5′ oligoadenylate synthetase family members (Oasl2, Oas3, Oasl1, Oas1a, Oas2, Oas1g), and additional interferon-stimulated genes such as IGTP and ISG20. These gene families have been extensively characterized in literature as components of innate immune signaling and host defense pathways. In other experimental and clinical contexts, reduced expression of interferon-stimulated gene programs has been associated with impaired antiviral and antitumor immune responses, and the therapeutic use of exogenous interferons in cancer treatment reflects the importance of these pathways in immune regulation (215, 216). Functional interferon signaling, pathogen defense, or therapeutic responsiveness was not directly assessed in the present study.

Interferon-stimulated genes (ISGs). The widespread repression of interferon-stimulated gene programs observed under chronic stimulation overlaps with transcriptional patterns previously reported in both chronic viral infections and cancer-associated immune regulatory states. In the literature, ISGs have been characterized as central components of innate immune signaling pathways involved in antibacterial and antiviral responses, including restriction of viral replication and modulation of pathogen virulence (217–219). Reduced expression of these gene programs has been associated in other experimental systems with increased susceptibility to viral infections such as hepatitis C virus, Ebola virus, influenza viruses, coronaviruses, and West Nile virus (220, 221).

Additional repressed transcripts observed here included Rtp4, a type I interferon receptor transporter, and Ly6e, both of which have been described in prior studies as contributors to antiviral immune responses across multiple immune cell types in models of HIV-1, SARS-CoV-2, and MERS-CoV infection (222–227). Repression of other IFN-inducible genes, including Xbp1, has similarly been reported in animal models to correlate with altered bacterial control during infection through pathways linking Toll-like receptor signaling and interferon responses (228, 229). Functional pathogen defense, infection susceptibility, or immune cell–specific responses were not directly assessed in the present study.

TLR and pathogen-recognition–associated genes. Transcriptomic analysis revealed sustained repression of CD14, a co-receptor for LPS–TLR4 signaling (230), and the C-type lectin receptor CLEC4E, a transmembrane receptor enriched in antigen-presenting myeloid lineages such as dendritic cells. In the literature, CLEC4E has been characterized as a pattern-recognition receptor capable of binding pathogen-associated and damage-associated molecular motifs, including lipopolysaccharide, through its carbohydrate recognition domain (231, 232). CLEC4 family members have also been described in other experimental systems as contributors to coordination between innate sensing pathways and adaptive immune responses, including effects on B- and T-lymphocyte activation (233). Notably, elevated CLEC4E expression has been reported as a feature of pro-inflammatory macrophage states, whereas reduced expression has been associated with immune-regulatory macrophage phenotypes in tumor-associated contexts (232, 234).

#### Additional interferon-associated antiviral gene programs

Chronic stimulation was associated with transcriptional repression of multiple interferon-associated antiviral gene families, including several Schlafen (SLFN) genes (Slfn1, Slfn2, Slfn4), TANK (TRAF-associated NF-κB activator), interferon-induced GTP-binding proteins (Mx), BST2, RSAD2 (viperin), RTP4, PCBP2, and the nuclear body protein Sp140. These genes have been extensively characterized in the literature as components of antiviral and innate immune signaling pathways across diverse experimental systems (235–249). In other disease models, altered expression of these factors has been associated with changes in immune regulation, pathogen control, and cancer-related immune environments, and SP140 in particular has been explored as a druggable target in inflammatory and oncologic contexts (e.g GSK761) (250–253). Conversely, depletion of SP140 compromises microbial defense and suppresses innate immune gene networks (254).

#### Autophagy-associated gene programs

Chronic stimulation in this study was associated with reduced transcription of several autophagy-linked interferon-stimulated genes, including Isg15, Isg20, and Sqstm1/p62. In the literature, these genes have been described as components of interferon-associated autophagy and protein quality-control pathways (255–259). Altered expression of SQSTM1/p62 has also been reported in diverse disease contexts, including cancer and age-related neurodegenerative disorders, where it has been linked to dysregulated protein turnover (260). In other experimental systems, plasmid-based strategies encoding p62/SQSTM1 (e.g., elenagen) have been explored as potential immunotherapeutic approaches for solid tumors (261, 262).

#### Lysozyme- and acute-phase–associated genes

Chronic stimulation in this study was associated with sustained transcriptional repression of antimicrobial-associated genes, including lysozyme 1 (Lyz1), lysozyme 2 (Lyz2), and serum amyloid A3 (Saa3). These genes have been characterized in the literature as components of innate antimicrobial and acute-phase response pathways, with roles in host defense against bacterial pathogens such as Staphylococcus, Streptococcus, and Escherichia coli (263–265). Reduced expression of these gene programs has been reported in other experimental systems in association with altered inflammatory and immune-regulatory states. Functional antimicrobial activity, mucosal barrier integrity, or infection susceptibility were not directly assessed in the present study.

### Category – tolerance: acute upregulation with diminished response in chronic stimulation

In a subset of responses, we see evidence of classical tolerance and reverse tolerance.

**Figure 13:**
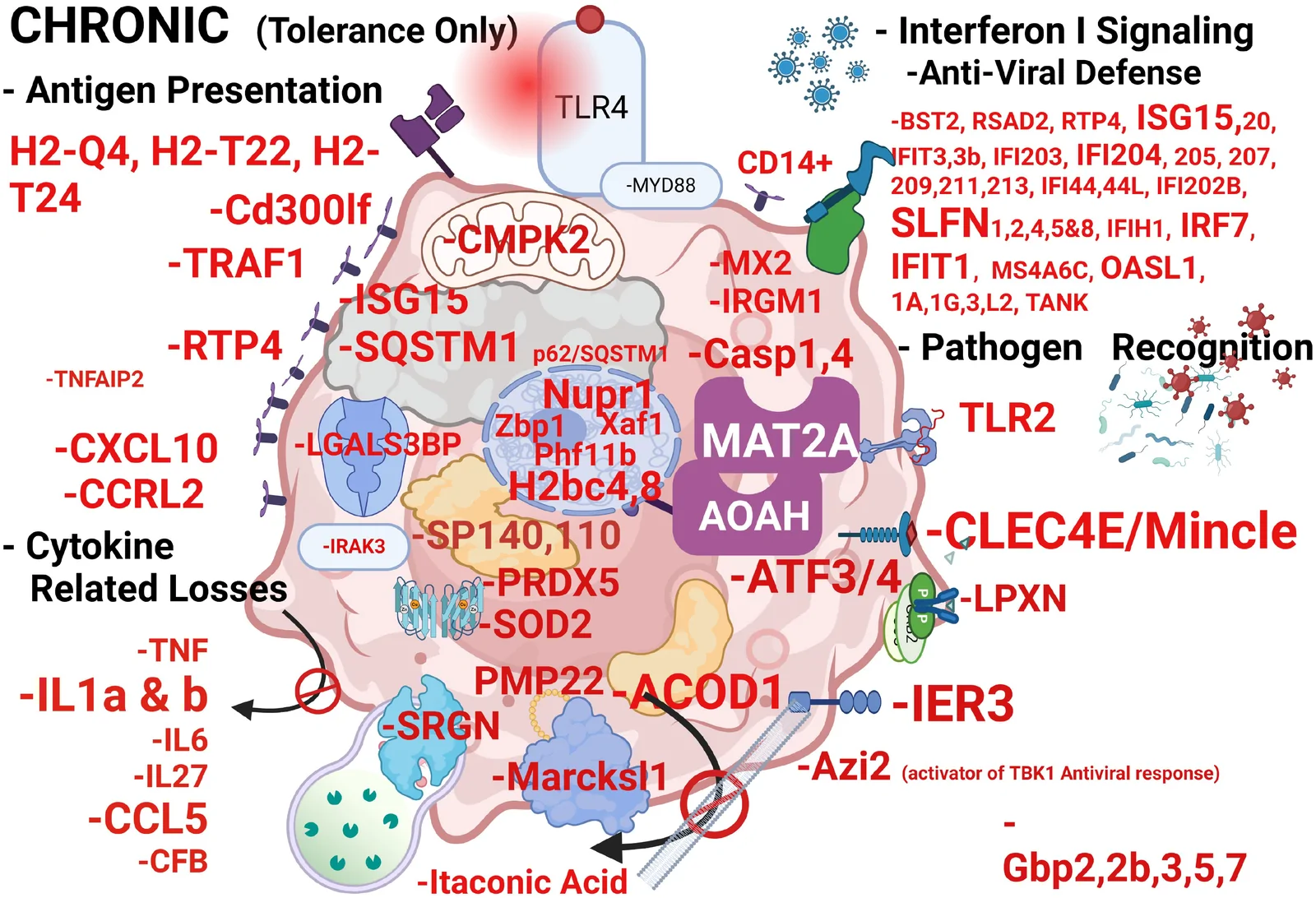
Tolerance effects on genes and pathways: acute induction with diminished response in chronic inflammation. The schematic illustrates the classical tolerance pattern, where genes strongly upregulated during acute LPS/TLR4 stimulation lose responsiveness under chronic exposure, ultimately returning toward baseline or becoming repressed. This tolerance trajectory dismantles antigen presentation (MHC-I loci), interferon/type I signaling (IFIT, IFI, OAS, SLFN families), cytokine networks (IL-1α/β, CCL5), pathogen recognition receptors (CD14, CLEC4E), and redox/detoxification systems, while weakening neutrophil homeostasis (Csf3) and complement pathways (Cfb). The cumulative effect is a macrophage transcriptome associated with losses in antigen presentation genes, blunted IFN responses, loss of inflammatory cytokine output, and cytotoxicity programs (SRGN, GBP family), converging on a well-described immune-tolerized, M2/TAM-like state. This tolerance-driven transcriptome shift reflects a loss of host defense against pathogens, undermines antitumor immunity, and fosters conditions that favor immune escape and tumor progression.

**Figure 13** illustrates a classical tolerance-associated transcriptional pattern in which genes acutely upregulated following LPS/TLR4 stimulation fail to sustain expression during prolonged exposure, resulting in progressive reduction or silencing at chronic time points. This behavior contrasts with DEG categories characterized by sustained induction or sustained repression. The tolerance pattern is defined by an early transcriptional peak during acute stimulation followed by diminished responsiveness during chronic stimulation and is observed across multiple immune- and inflammation-associated gene programs.

Chronic stimulation was associated with transcriptional repression of Cfb (complement factor B), a component of the alternative complement pathway. In the literature, altered CFB expression has been reported in association with immune-regulatory states and has been linked to clinical outcomes in lung cancer as well as to changes in innate immune signaling in other experimental systems (336) (266, 267).

TNF- and IL-1–associated gene programs. Chronic stimulation was associated with altered transcriptional patterns across multiple TNF- and IL-1–related signaling components. Several TNF-associated genes exhibited tolerance-like behavior, including Traf1, Tnfaip2, Traf2, Tnfrsf22, Tnfrsf23, and Tnfrsf26, whereas Tnfrsf1b (TNFR2) remained modestly elevated relative to the resting baseline. In the literature, TNFR2 expression has been associated with immune-regulatory environments, including myeloid-derived suppressor cell–rich contexts (268–270).

Tolerance-associated transcriptional repression also extended to IL-1/TLR-related genes, with near-complete reduction of Il1a, Il1b, and Irak3, while Il1rn (IL-1 receptor antagonist) remained elevated relative to baseline. Similar transcriptional patterns involving IL-1 pathway components have been reported in chronic inflammatory and cancer-associated immune-regulatory settings, where altered IL-1 signaling has been linked to changes in immune responsiveness and disease progression (271, 272).

#### ATF family members and serglycin

Chronic stimulation was associated with pronounced transcriptional repression of Atf3, Atf4, and serglycin (Srgn). Reduced expression of ATF family transcription factors has previously been identified through bioinformatic analyses of aggressive human cancers and chronic inflammatory states (273). Serglycin is a proteoglycan that has been extensively characterized in the literature as a component of granule biology in multiple immune cell types, including macrophages, natural killer cells, mast cells, neutrophils, and cytotoxic T lymphocytes, where it is involved in the storage of cytotoxic mediators (274–276). In animal models, loss of Srgn has been associated with altered immune cell composition and impaired immune responses in the context of cancer and viral infection (276–280).

In parallel, chronic stimulation was associated with transcriptional repression of TYROBP, an adaptor protein involved in immune receptor signaling pathways. In the literature, altered TYROBP expression has been reported in association with immune-regulatory states and has been linked to cancer progression and survival outcomes in specific malignancies, including osteosarcoma (281, 282). Functional immune activation and clinical prognosis were not directly evaluated in the present study.

### Reverse tolerance: repression in acute, restored to resting baseline in chronic stimulation

**Figure 14:**
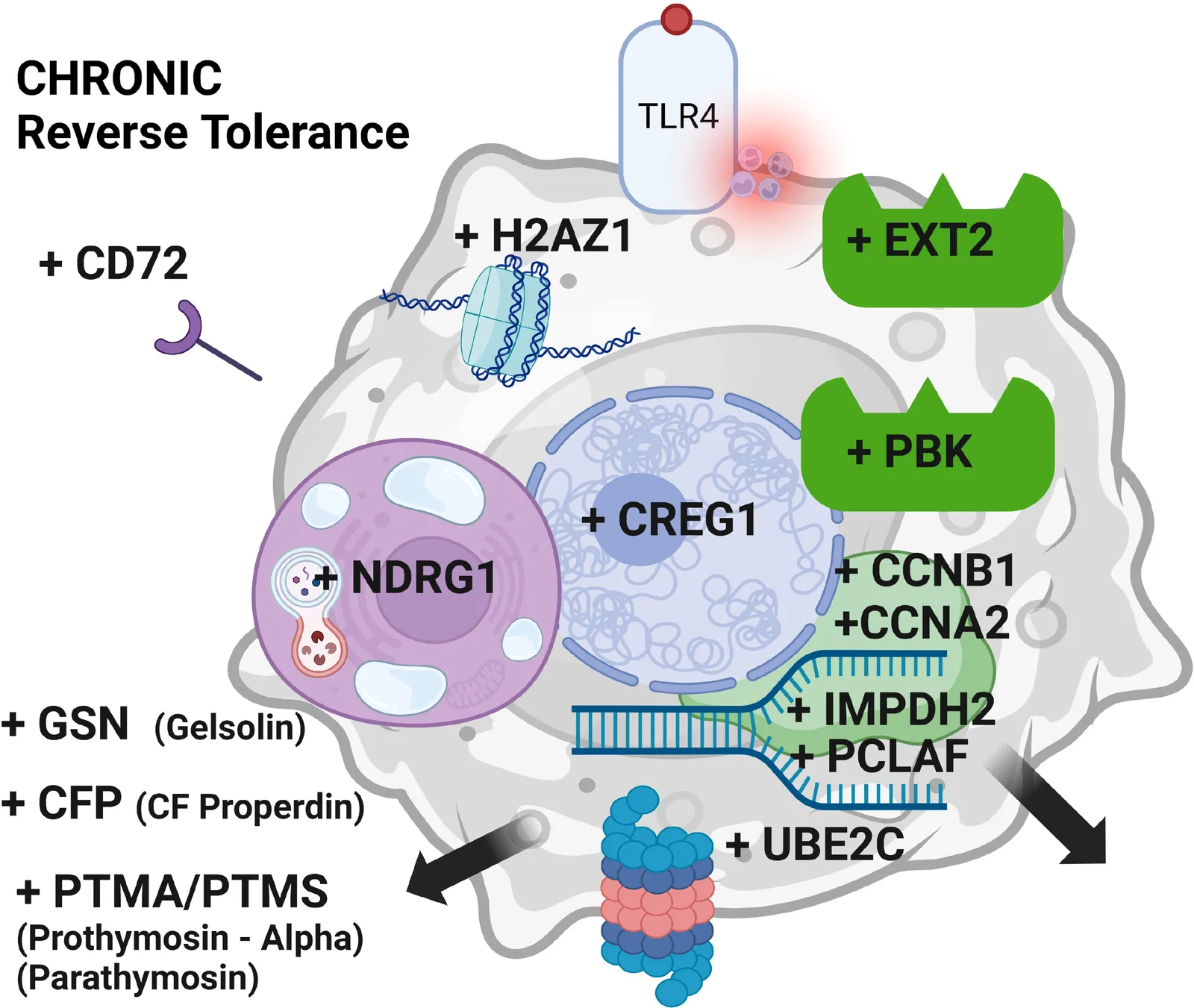
Reverse tolerance transcriptional trajectory under chronic stimulation.The schematic depicts transcripts that are transiently repressed during acute LPS/TLR4 stimulation and subsequently return to resting baseline expression under chronic exposure. This pattern, referred to here as “reverse tolerance,” differs from classical tolerance by exhibiting recovery of expression at later time points. Transcripts following this trajectory were enriched for gene sets annotated to chromatin remodeling, DNA replication, and cell cycle–associated processes. Among these, PTMA (prothymosin alpha) was identified as a prominently restored transcript. In the literature, PTMA expression has been reported in association with cancer-related and inflammatory contexts; however, functional roles, disease relevance, or therapeutic implications were not evaluated in the present study.

**Figure 14** illustrates a distinct subset of transcripts that follow a reverse tolerance transcriptional trajectory, characterized by repression during acute inflammation and restoration to resting baseline expression under chronic conditions. This pattern differs from classical tolerance, in which transcriptional responses progressively diminish over time. Genes exhibiting reverse tolerance were enriched for pathways annotated to chromatin remodeling, DNA replication, and cell cycle–associated processes. The recovery of expression across these gene sets highlights a reproducible transcriptional behavior observed during prolonged stimulation.

Reverse Tolerance – PTMA as a Key Marker.

The category of reverse tolerance is defined by transcripts that are repressed during acute inflammation and subsequently return to resting baseline expression under chronic conditions. Among the most prominently restored transcripts observed in this study was PTMA (prothymosin alpha). In the literature, PTMA has been described as an immune- and stress-associated protein and has been reported in association with cancer-related and inflammatory contexts. Prior studies have linked PTMA expression to lipid droplet–associated processes in colon cancer tissue and have reported elevated PTMA levels in esophageal cancer relative to adjacent normal tissue (283, 284). PTMA has also been discussed in the literature as a molecule of interest in immune-related diseases, including cancer, chronic inflammation, and sepsis (285).

## Study limitations

While this study provides a high-resolution map of the transcriptional transitions from acute activation to chronic tolerance, certain limitations must be acknowledged. First, the use of the immortalized RAW 264.7 macrophage cell line was a deliberate methodological choice to ensure experimental stability and reproducibility over an extended 11-day period. Primary myeloid cells, such as bone marrow-derived macrophages (BMDMs), are notoriously prone to phenotypic drift, growth factor dependency, and declining viability when maintained in long-term culture. By utilizing a stable, fully viable model combined with a rigorous 18-hour media exchange protocol, we were able to resolve a complex eight-phase gene trajectory that might otherwise be obscured by the biological noise and “culture aging” inherent in primary cell systems over such a duration.

Second, although the RAW 264.7 model serves as a robust tool for investigating TLR4-mediated signaling pathways, it is an *in vitro* system that lacks the intricate three-dimensional architecture and multifaceted cytokine signaling of an *in vivo* tumor microenvironment. Nevertheless, the striking alignment between our 11-day transcriptional signatures—specifically the upregulation of immune checkpoints (PD-L1, TIM-3, CD73) and the loss of MHC I/II antigen presentation—and the phenotypes observed in clinical tumor-associated macrophages (TAMs) underscores the biological relevance of this model. This study establishes a foundational “transcriptional blueprint”; future research will focus on validating these specific markers and kinetic phases in primary human cells and complex *in vivo* models to further elucidate the therapeutic potential of reversing chronic immune suppression.

## Conclusion

This study demonstrates that chronic inflammatory stimulation is associated with coordinated and reproducible transcriptional reprogramming in macrophages that extends beyond sustained activation alone. Distinct patterns of classical tolerance, reverse tolerance, and sustained induction or repression define a structured transcriptional landscape under prolonged stimulation. These patterns include concerted repression of gene programs annotated to antigen presentation, interferon-associated responses, and cytotoxicity-related pathways, alongside sustained expression of immune-regulatory, stress-associated, and metabolic genes such as PD-L1, TIM-3, SOCS3, S100A8, and cathepsins. Collectively, the resulting transcriptomic profile is dominated by negative feedback and aligns with immune-regulatory signaling environments described in the literature in chronic inflammation and cancer-associated contexts.

Taken together, these data support a model in which tolerance reflects a coordinated transcriptional program rather than a passive loss of responsiveness, providing a framework for future studies aimed at dissecting how chronic inflammatory signaling reshapes immune regulation.

## Data Availability

The data presented in the study are deposited in the NCBI Gene Expression Omnibus (GEO) repository, accession number GSE263901.

## Supplementary material

Supplementary File S1 **Table 1A**. Enriched biological processes and pathways are downregulated in chronic inflammation. Functional enrichment analysis of differentially expressed genes (DEGs) suppressed during chronic inflammation, performed using GO terms, KEGG pathways, and InterPro annotations. The table reports the database and ontology ID, associated term, percentage of annotated genes, gene list, fold enrichment, and adjusted Benjamini–Hochberg p-values. Highlighted pathways include strong suppression of viral defense responses, tumor necrosis factor production, type I interferon signaling, NOD signaling, antigen presentation, and mitochondrial metabolism, all consistent with transcriptional repression of pathways required for innate and adaptive immune surveillance. **Table 1B**. Enriched biological processes and pathways are upregulated in chronic inflammation. Functional enrichment of DEGs increased during chronic stimulation, spanning GO (BP/CC/MF), KEGG, InterPro, SMART/UP features. Columns report database/ontology ID and term, percent of annotated genes, gene list, fold enrichment, and Benjamini–Hochberg adjusted *p*-values. Dominant themes include cytokine/cytokine-receptor signaling (chemokines CCL2/4/6/7/9/12; JAK–STAT: JAK1/2, STAT1/2, SOCS3), leukocyte chemotaxis (neutrophil/eosinophil/monocyte), FcγR-mediated phagocytosis, lysosomal/vesicular modules (V-ATPase, AP-3), cell–cell junction/adhesion, TNF/IL-1 responses, and NF-κB/MAPK regulators, small GTPase/Rho signaling, apoptosis/Bcl-2 family features, lipid handling (fatty-acid transport/sphingolipids), and ferroptosis components. This pattern is consistent with a coordinated regulatory inflammatory program characteristic of chronic activation.

Supplementary Table 2 This table summarizes transcriptome-wide differential expression analysis comparing Control vs LPS (Acute), Control vs T2, Control vs T3, and LPS vs T2/T3 conditions. For each gene, the following are reported: • Description/Gene ID/Gene Name: Annotation of the transcript, including official symbol and Ensembl identifier. • Differential analysis results: For each pairwise contrast (Control vs LPS, Control vs T2, Control vs T3, LPS vs T2, and LPS vs T3), values include:   • *baseMean* (mean normalized expression across samples),   • *log2FoldChange* (direction and magnitude of regulation),   • *FC* (linear fold change),   • *p-value* (significance of differential expression). • Mean FPKM: Average normalized expression values (FPKM) for each group: Control, LPS (Acute), T2, and T3. • General Pattern: Categorization of overall expression trajectory across conditions (e.g., High Acute Sustained, High in Chronic, Tolerance-like suppression, Recovery phase, etc.). • Significant Pattern: Denotes whether the observed profile is supported by statistically significant changes between groups in a specific pattern. Asterisks indicate confidence levels (*, **, ***). • Color coding: FPKM expression patterns are color-coded to highlight genes fitting various patterns where significance of that pattern is determined by adherence to expected patterns across conditions.

## Glossary

ACOD1: aconitate decarboxylase 1
ATP: adenosine triphosphate
CARD19: caspase recruitment domain family member 19
CCL: chemokine (C-C motif) ligand
CD36: fatty-acid translocase
COX2: cyclooxygenase-2
CTL: cytotoxic T lymphocyte
CXCL: chemokine (C-X-C motif) ligand
DAVID: Database for Annotation, Visualization, and Integrated Discovery
DEG: differentially expressed gene
ELISA: enzyme-linked immunosorbent assay
FABP: fatty-acid binding protein
FPKM: fragments per kilobase of transcript per million mapped reads
GO: Gene Ontology
IFI: interferon-induced protein
IFIT: interferon-induced transcript
IFN-I: type I interferon
iNOS: inducible nitric oxide synthase
ISG: interferon-stimulated gene
IL: interleukin
JAK/STAT: Janus kinase/signal transducer and activator of transcription
KEGG: Kyoto Encyclopedia of Genes and Genomes
LPS: lipopolysaccharide
MAMP: microbe-associated molecular pattern
MAPK: mitogen-activated protein kinase
MDSC: myeloid-derived suppressor cell
MHC: major histocompatibility complex
NF-κB: nuclear factor kappa-light-chain-enhancer of activated B cells
NK: natural killer cell
NOD: nucleotide-binding oligomerization domain
OAS: 2′-5′-oligoadenylate synthetase
OXPHOS: oxidative phosphorylation
PD-L1: programmed death-ligand 1
SOCS: suppressor of cytokine signaling
TAM: tumor-associated macrophage
TAN: tumor-associated neutrophil
TLR4: Toll-like receptor 4
TNF: tumor necrosis factor
Treg: regulatory T cell.
